# Supplementary material for: Precision of Readout at the hunchback Gene: Analyzing Short Transcription Time Traces in Living Fly Embryos
Source: PLoS Comput Biol. 2016 Dec 12;12(12):e1005256. doi: 10.1371/journal.pcbi.1005256 (PMC5152799; doi:10.1371/journal.pcbi.1005256)
Supplement: S1 Text — (PDF) [file pcbi.1005256.s001.pdf]

Supporting Information for: Precision of readout at the hunchback gene

Jonathan Desponds[1,2,3], Huy Tran[1,2,3], Teresa Ferraro[1,2,3], Tanguy Lucas[2,3,4], Carmina Perez Romero[5], Aurelien Guillou[2,3,4], Cecile Fradin[5], Mathieu Coppey[2,3,4], Nathalie Dostatni[2,3,4] and Aleksandra M. Walczak[1,2,31]
1

[1] *Ecole Normale Superieure, PSL Research University, Paris, France*
[2] *Sorbonne Universités, UPMC Univ Paris 06, Paris, France*
[3] *UMR3664/UMR168/UMR8549, CNRS, Paris, France*
[4] *Institut Curie, PSL Research University, Paris, France*
[5] *McMaster University, Canada*
(Dated: November 23, 2016)

I. AUTOCORRELATION ANALYSIS

A. Basic setup and data preprocessing

All raw data are made available via the following link:
`http://xfer.curie.fr/get/GmJzLUbF1JU/mov.zip`
In case of difficulty downloading the data, please contact us directly. The raw data produced experimentally is a fluorescent signal $I(t)$ measured at discrete times corresponding to the sampling time frame of the movie (see SIFig. 6 for examples of traces). At each locus and at each time point it is the sum of the background signal and a number of fluorescent molecules attached to loops formed by the mRNA. Each loop contributes to the signal by a constant I_0. This constant is unknown and can vary from trace to trace due to noise in the experimental setup and the variability in the locations of the nuclei in the embryo. All models are written for the renormalized signal $F(t) = I(t)/I_0$.

Because the fluorescent signal is produced by discrete polymerases that travel down the gene, we divide the gene into chunks of 150 base pairs, a length that corresponds to the irreducible space occupied by a polymerase on the gene (Fig. 4 in the main text). The positions the polymerase can occupy on the gene are labeled by an index $1 \leq i \leq r$. The number of MS2 loops that have been formed by a polymerase that has reached a given position depends only on the MS2 gene construct and we define a deterministic function L_i for the whole length of the gene that describes the number of MS2 loops that have been produced by a polymerase at position i. In practice the exact number of loops is not an integer and varies from base pair to base pair so we take L_i as the average number of loops at this polymerase position (see Fig. 4 in the main text).

When the gene is fully loaded with polymerases (the number of polymerases is equal to the length of the gene divided by 150 bp), the fluorescence intensity is $I(t) = I_0 \sum_i^r L_i$. Assuming that the maximum of the signal over the whole trace is a good approximation for the fully loaded value we can determine I_0 and renormalize the data. In practice, since we see variability in the expressed signal in different nuclei at the same position, we are not sure the fully loaded polymerase scenario occurs in each nuclei, so we take the mean of the maximum intensity values in the anterior. We use this renormalized fluorescence signal to infer the parameters of the dynamics.

The experimental data is analyzed assuming the system is in steady state and does not take into account the initial activation period after mitosis, and the end of the trace when the gene is deactivated before mitosis. We take only a window of the traces where the mean spot intensity in all traces is stable (see SIFig. 2 for an example). As the duration of the interphase differs slightly between embryos, we use a different steady state window for each embryo as summarized in Table I.

In all models based on a stochastic gene switching (so all models except the Poisson-like model) we assume that the gene can be in several states with only two effective transcription rates: a non zero transcription rate in the ON state and an basal production rate equal to zero in the OFF state. When the gene is ON the polymerase loads at a maximal rate set by clearing of the binding site by the previous polymerase, which is one polymerase every 6 seconds (calculated as the irreducible polymerase length along the gene 150 bp divided by the polymerase speed, $v = 25bp/s$). The state of the gene is described by a stochastic process $X(t)$ that is equal to 1 when the gene loads polymerase (i.e is ON) and 0 when the gene is OFF (see Fig. 1B in the main text). Once the polymerase is loaded its path is assumed to be deterministic with constant speed.

The gene can be described by the locations where there is a polymerase: we define $a(i, t)$ as a function of time t and position $1 \leq i \leq r$ that is equal to 1 if there is polymerase at position i at time t and 0 otherwise (see Fig. 1D in the main text). The fluorescence signal is then a convolution of the polymerase position, $a(i, t)$, and the details of

the loop design of the MS2 construct, L_i:

$$F(t) = \sum_{i=1}^{r} L_i a(i,t), \tag{1}$$

and the polymerase position can easily be translated back to the gene state through the deterministic relation, $a(i,t) = X(t-i)$ (see Fig. 3D in the main text for the form of L_i). This disruption is exact for a system with a discrete regulatory process and a discrete time step equal to the polymerase time step. Unfortunately, the moments in time when the gene switches are not necessarily multiples of the natural coarse graining steps of the system (the polymerase time step and its equivalent length) so it is necessary to introduce a continuous time in the system. We will present results for both the discrete and continuous time models. The continuous description is valid in the limit where the typical time spent by the gene in each state is long compared to the polymerase step or equivalently the gene switching constants are small compared to $1/6\ \mathrm{s}^{-1}$. See SI Section I B for a more detailed argument.

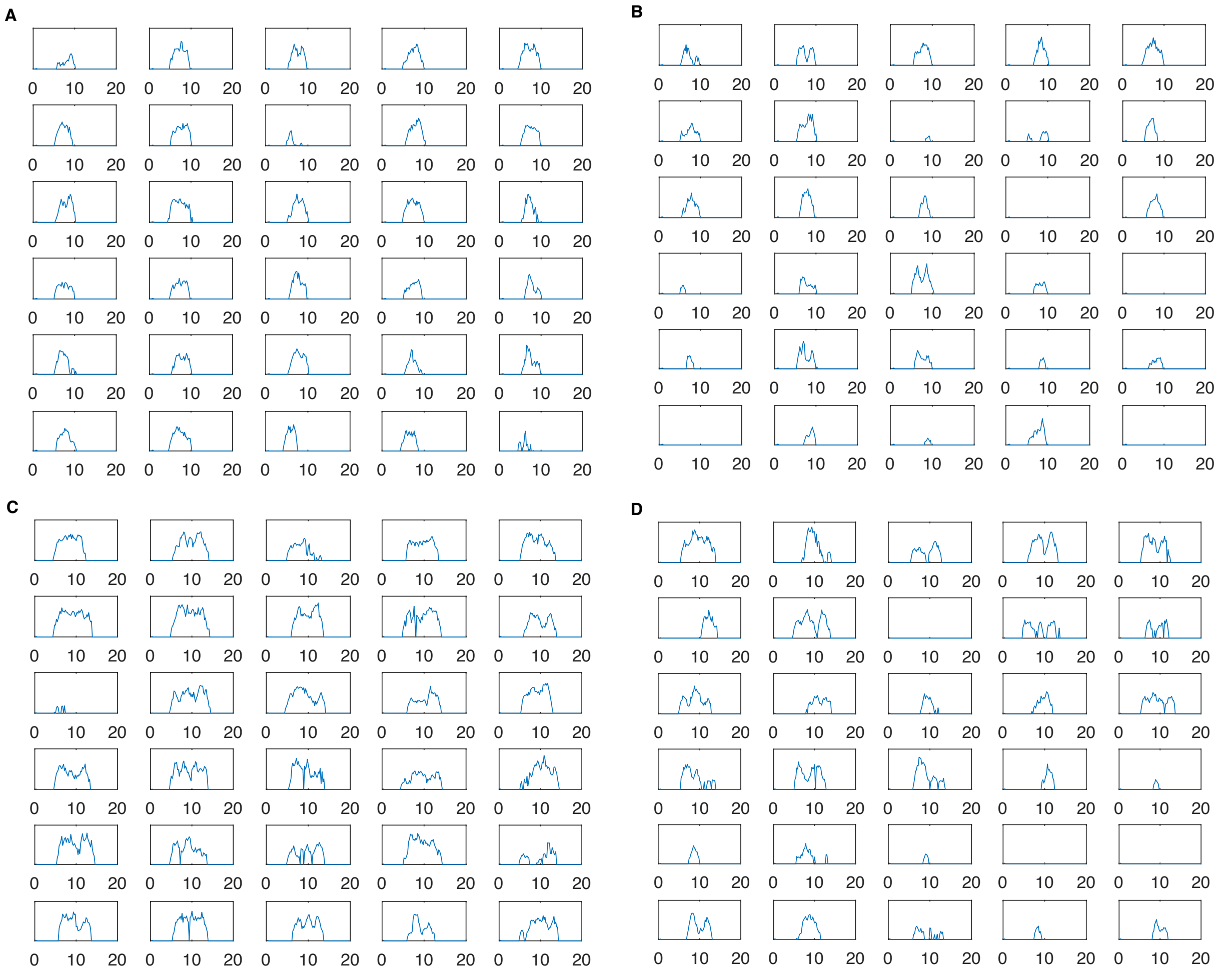

FIG. 1: **Examples of individual spot intensity over time**. Consecutively shown are the traces in (A) Cycle 12, Anterior, (B) Cycle 12, Boundary (C) Cycle 13, Anterior, (D) Cycle 13, Boundary. The x axis is time in minute and y axis is the spot intensity in AU.

B. The two-state model

In this section we derive the equations required for the inference of the dynamics under the assumption that the gene can be in two states: ON or OFF, represented by a two dimensional vector $x(t) = [x_{\mathrm{on}}(t), x_{\mathrm{off}}(t)]$. $x_{\mathrm{on}}(t)$ is the probability of the gene to be ON and $x_{\mathrm{off}}(t)$ is the probability of the gene to be OFF. $x_{\mathrm{on}}(t)$ is the average over traces

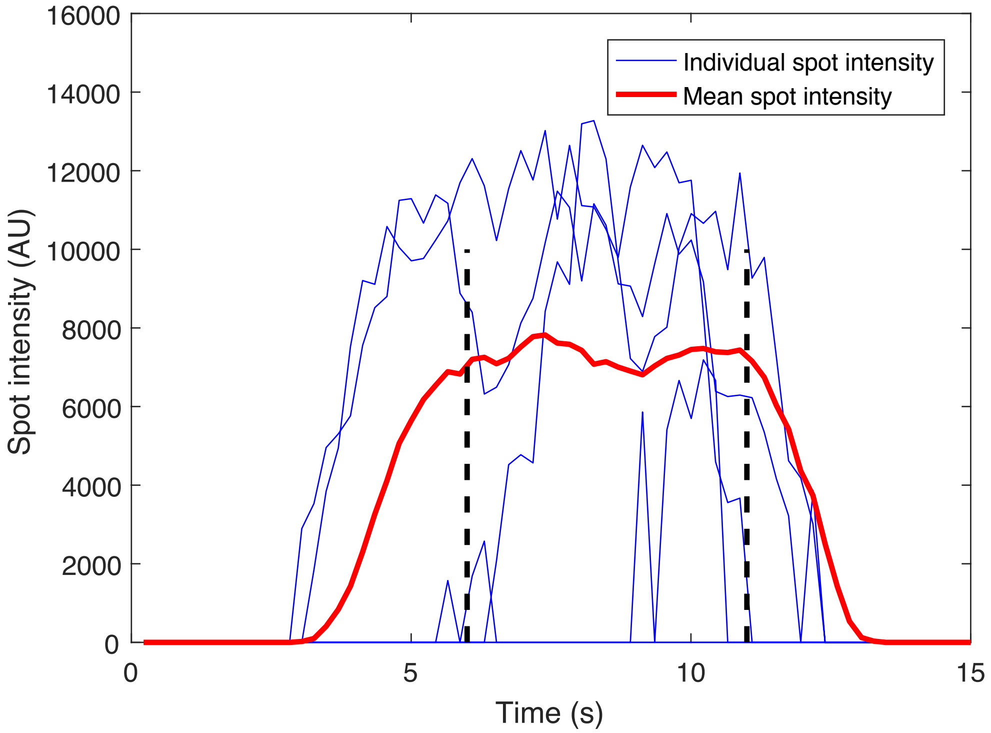

FIG. 2: **Data calibration**. Shown are examples of 5 (out of 154) individual traces (blue) taken from embryo 1, cycle 13. Also shown is the mean spot intensity over time of all traces (red). The steady state window is chosen to be from the 6th minute to the 11th minute (dashed lines).

	beginning (s)	end (s)	interphase duration (s)
Embryo 1 - cc 12	278	391	652
Embryo 1 - cc 13	318	546	796
Embryo 2 - cc 12	281	393	570
Embryo 2 - cc 13	278	484	695
Embryo 3 - cc 12	297	408	676
Embryo 3 - cc 13	330	587	824
Embryo 4 - cc 12	261	515	616
Embryo 4 - cc 13	321	529	751

TABLE I: The steady-state window for the autocorrelation analysis: Shown for each embryo and cell cycle are the beginning and ending times of the steady state window, and the duration of interphase.

of the random variable $X(t)$ depicted in Fig. 1B of the main text. We assume that the switching times between the two are exponentially distributed:

$$\partial_t \begin{pmatrix} x_{\text{on}} \\ x_{\text{off}} \end{pmatrix} = \begin{pmatrix} -k_{\text{off}} & k_{\text{on}} \\ k_{\text{off}} & -k_{\text{on}} \end{pmatrix} \begin{pmatrix} x_{\text{on}} \\ x_{\text{off}} \end{pmatrix}. \tag{2}$$

The steady state probability to be ON is $P_{\text{on}} = x_{\text{on}}(t = \infty) = 1/T \sum_t x_{\text{on}}(t)$, where T is the duration of the steady state window in Fig. 2, and is:

$$\frac{k_{\text{on}}}{k_{\text{off}}} = \frac{P_{\text{on}}}{1 - P_{\text{on}}}. \tag{3}$$

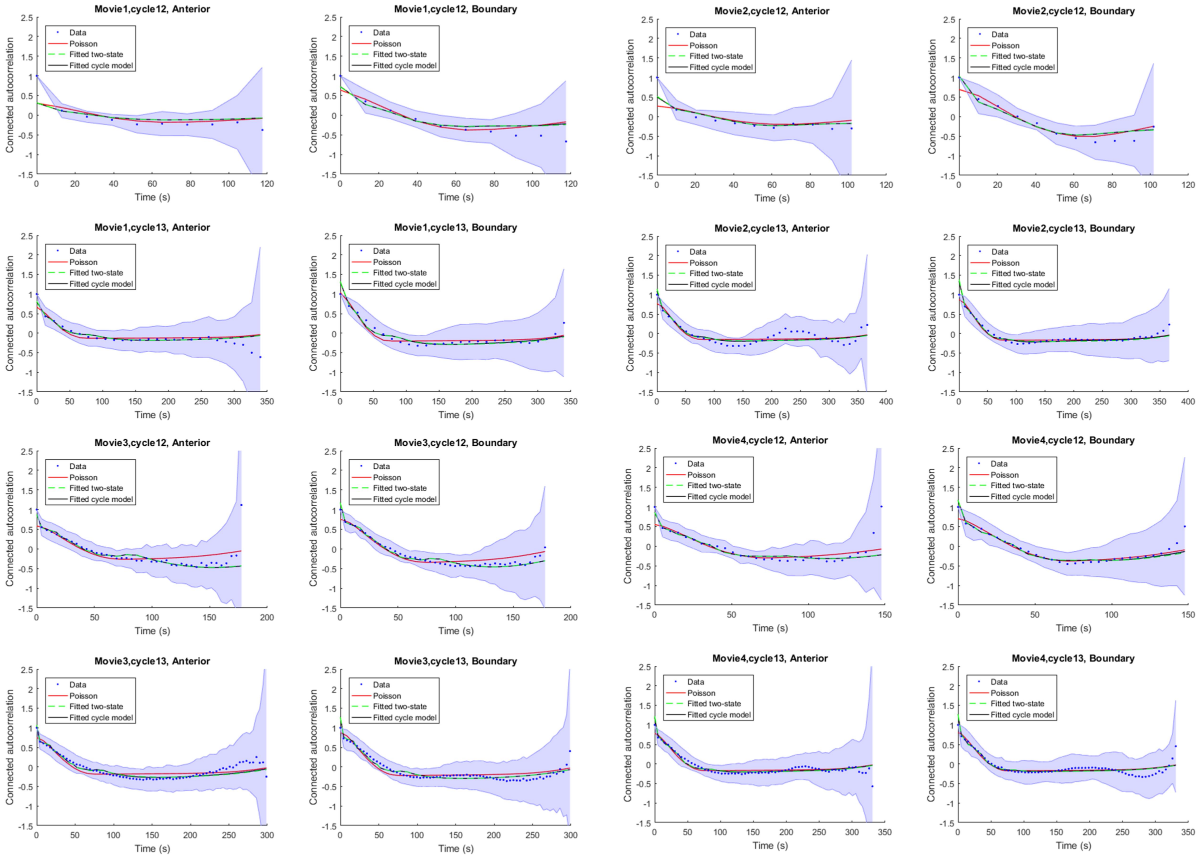

FIG. 3: **Fits of the autocorrelation function**. The empirical autocorrelation function (blue dots) for both the anterior and boundary regions in all four embryos is fit using the autocorrelation function with the finite size corrections for the Poisson-like model (red lines), two-state model (green lines) and three-state cycle model (black lines).

kon$(1/s)$	mov1	mov2	mov3	mov4
12A	0.078	0.056	0.009	0.023
12B	0.004	0.005	0.003	0.011
13A	0.017	0.020	0.014	0.021
13B	0.004	0.006	0.004	0.005

TABLE II: The inferred $k_{\rm on}$ rates from the autocorrelation approach assuming a two-state model for the four embryos and cell cycle 12 and 13, in the anterior and boundary.

We learn $P_{\rm on}$ from Eq. 1 in the main text:

$$\langle F \rangle = P_{\rm on} \sum_{i=1}^{r} L_i. \tag{4}$$

and use it to obtain the ratio of the switching rates from Eq. 3.

The autocorrelation function is:

$$\langle F(t)F(s) \rangle = \sum_{i=1}^{r} \sum_{j=1}^{r} L_i L_j \langle a(i,t)a(j,s) \rangle, \tag{5}$$

where the brackets are an average over traces (different realizations of the random process). We define $A(t-i, s-j) = 1/x_{\rm on}(s-j)\langle a(i,t)a(j,s)\rangle$ – the probability that the polymerase is at position i and time t given that there was a polymerase at position j at time s (here we assume that $t - i \geq s - j$). Using the deterministic relation between the

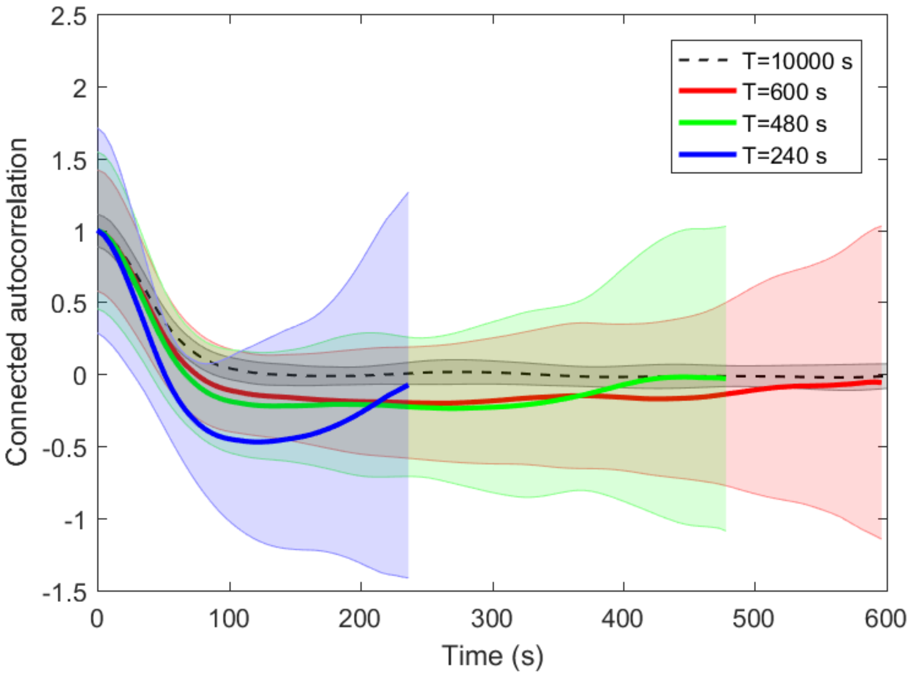

FIG. 4: **Example of the connected autocorrelation function for the two state model calculated for different trace lengths T.** The shaded areas denote the standard variation over 500 simulated traces. The switching rates $k_{\rm on} = k_{\rm off} = 0.01 s^{-1}$.

koff$(1/s)$	mov1	mov2	mov3	mov4
12A	0.060	0.088	0.008	0.019
12B	0.020	0.034	0.021	0.051
13A	0.018	0.031	0.016	0.027
13B	0.031	0.054	0.031	0.064

TABLE III: The inferred $k_{\rm off}$ rates from the autocorrelation approach assuming a two-state model for the four embryos and cell cycle 12 and 13, in the anterior and boundary.

polymerase position at a given time $a(i,t)$ and the probability to be on at an earlier time $X(t-i)$, $A(t-i,s-j)$ is equivalent to the probability that the gene in ON at time $t-i$ given that is was ON at time $s-j$:

$$A(t-i,s-j) = x_{\rm on}(t-i|\text{ ON at time } s-j). \tag{6}$$

Plugging the expression into Eq. 5 we obtain Eq. 2 in the main text:

$$\langle F(t)F(s)\rangle = \sum_{i=1}^{r}\sum_{j=1}^{r} L_i L_j x_{\rm on}(\min(s-j,t-i))A(t-i,s-j). \tag{7}$$

In steady state the system is translationally invariant $A(t-i,s-j) = A(|t-i-s-j|)$ and for brevity we will denote it as $A(n)$ - the probability that the gene is ON at time n, given that it was ON at time 0. To find A_n we need to solve for $x(t)$:

$$\partial_t x(t) = (T - \mathbb{1})x(t), \tag{8}$$

where $T - \mathbb{1}$ is given by Eq. 2 and calculate the expectation value that the gene is ON at time t given in was ON initially:

$$A_n = \begin{pmatrix} 1 & 0 \end{pmatrix} e^{n(T-\mathbb{1})} \begin{pmatrix} 1 \\ 0 \end{pmatrix}. \tag{9}$$

Eq. 9 is correct in a continuous time model. Its discrete time equivalent is

$$A_n = \begin{pmatrix} 1 & 0 \end{pmatrix} T^n \begin{pmatrix} 1 \\ 0 \end{pmatrix}. \tag{10}$$

In the limit of k_{on} and k_{off} much smaller than the polymerase step they are also much smaller than 1 and $e^{n(T-\mathbb{1})} \simeq \mathbb{1} + n(T-\mathbb{1}) \simeq (\mathbb{1} + (T-\mathbb{1}))^n$. In this limit the continuous and discrete time descriptions of Eq. 9 and Eq. 10 are equal.

The eigenvalues of $T - \mathbb{1}$ are $[1, \delta]$, where $\delta = 1 - k_{\text{on}} - k_{\text{off}}$ with corresponding eigenfunctions:

$$\begin{pmatrix} P_{\text{on}} \\ P_{\text{off}} \end{pmatrix}, \begin{pmatrix} 1 \\ -1 \end{pmatrix}. \tag{11}$$

The transition matrix T is

$$T = \frac{1}{P_{\text{on}} + P_{\text{off}}} \begin{pmatrix} P_{\text{on}} & 1 \\ P_{off} & -1 \end{pmatrix} \begin{pmatrix} 1 & 0 \\ 0 & \delta \end{pmatrix} \begin{pmatrix} 1 & 1 \\ P_{off} & -P_{\text{on}} \end{pmatrix} \tag{12}$$

and

$$e^{n(T-\mathbb{1})} = \begin{pmatrix} P_{\text{on}} + e^{n(\delta-1)} P_{\text{off}} & P_{\text{on}} - e^{n(\delta-1)} P_{\text{on}} \\ P_{off} - e^{n(\delta-1)} P_{\text{off}} & P_{\text{off}} + e^{n(\delta-1)} P_{\text{on}} \end{pmatrix} \tag{13}$$

resulting in

$$A_n = P_{\text{on}} + e^{n(\delta-1)} P_{\text{off}}. \tag{14}$$

In steady state $x_{\text{on}}(s-j) = P_{\text{on}}$ and the connected autocorrelation is:

$$\tilde{C}_\tau = < F(t)F(t+\tau) > - < F(t) >^2 = \sum_{i=1}^{r} \sum_{j=1}^{r} L_i L_j P_{\text{on}} P_{\text{off}} e^{|\tau - j + i|(\delta-1)}. \tag{15}$$

Since we already know the ratio of the rates from P_{on}, inferring δ using Eq. 15 determines k_{on} and k_{off}.

C. Computing out of steady state

The autocorrelation approach can be generalized to a case when the system is out of steady state, when the autocorrelation function explicitly depends on the two time points and not only on their difference. During mitosis the gene is OFF and then gets turned ON in early interphase. Motivated by the *hunchback* expression we will present the calculation assuming the gene is initially OFF, but it is generalizable to any other initial condition. Assuming $t - i < s - j$, we want to calculate the probability that the polymerase is at position i at time t, given that it was at position j at time s. Since the gene is initially OFF, we need to calculate the probability that the gene is ON at time $t - i$. The autocorrelation function of the polymerase position is:

$$\langle a_i(t) a_j(s) \rangle = \begin{pmatrix} 1 & 0 \end{pmatrix} e^{(s-t+i-j)(T-\mathbb{1})} \begin{pmatrix} 1 \\ 0 \end{pmatrix} \begin{pmatrix} 1 & 0 \end{pmatrix} e^{(t-i)(T-\mathbb{1})} \begin{pmatrix} 0 \\ 1 \end{pmatrix}. \tag{16}$$

Using Eq. 14 and

$$\begin{pmatrix} 1 & 0 \end{pmatrix} e^{n(T-\mathbb{1})} \begin{pmatrix} 0 \\ 1 \end{pmatrix} = P_{\text{on}}(1 - e^{n(\delta-1)}), \tag{17}$$

we obtain:

$$\langle F(t)F(s) \rangle = \sum_{i=1}^{r} \sum_{j=1}^{r} L_i L_j P_{\text{on}} (1 - e^{(\delta-1)\min(t-i, s-j)}) (P_{\text{on}} + P_{\text{off}} e^{|s-j-t+i|(\delta-1)}). \tag{18}$$

D. Multiple off states

The calculations presented in Appendix I B can be extended to models that include more OFF or ON states as long there are only two production states for the mRNA: one enhanced and one basal production state. The transition matrix T will then be of higher dimension and in practice should be (and has to be for dimensions larger than 3) diagonalized numerically. The exact analytical solution for the autocorrelation function is still valid written in terms of the powers of T.

E. Generalized multi step model

A gene with many OFF states can also be described using a reduced model with two effective gene expression states ON and OFF, where the times of transitions between these two state are not exponential but follow a peaked distribution approximated by a Gamma distribution. The Gamma distribution describes an effective transition over many irreversible transitions between a series of OFF states:

$$\Gamma_{\alpha,\beta}(x) = \frac{\beta^{\alpha}}{\Gamma(\alpha)} x^{\alpha-1} e^{-\beta x}, \tag{19}$$

where β is the scale parameter, α is the shape parameter, and $\Gamma(\alpha)$ is the gamma function. The mean time spent in the OFF state is $1/k_{\text{on}}^{\text{eff}} = \alpha/\beta$, so the probability for the gene to be in the ON state is:

$$P_{\text{on}} = \frac{k_{\text{on}}^{\text{eff}}}{k_{\text{on}}^{\text{eff}} + k_{\text{off}}} = \frac{1}{1 + \alpha k_{\text{off}}/\beta}. \tag{20}$$

This model has three parameters, regardless of the number of OFF states, and using Eq. 1 of the main text reduces the number of parameters to two, which greatly simplifies the inference. The remaining two parameters are learned from the autocorrelation function in Eq. 5, which formally has the same form as Eq. 7:

$$\langle F(t)F(s)\rangle = \sum_{m=1}^{r} \sum_{n=1}^{r} L_m L_n x_{\text{on}}(\min(t-m, s-n)) A_\Gamma(|s-n-t+m|), \tag{21}$$

but $A_\Gamma(|s-t+m-n|)$ is now the two-point correlator of a non-Markovian process. We limit our presentation to the steady state, but the calculation generalizes to out of steady state systems.

We cannot solve the problem in real space, but we compute the Fourier transform of the autocorrelation function of the fluorescence signal:

$$\hat{C}(\xi) = \int_{-\infty}^{+\infty} d\tau (\langle F(t)F(t+\tau)\rangle - \langle F(t)\rangle^2) e^{-2i\pi\tau\xi}, \tag{22}$$

which using Eq. 5

$$\hat{C}(\xi) = P_{\text{on}} \sum_{m,n} L_m L_n 2\Re\left[e^{-2i\pi(m-n)} \hat{A}_\Gamma^*(\xi)\right] \tag{23}$$

we reduce to calculating

$$\hat{A}_\Gamma^*(\xi) = \int_0^{+\infty} dt e^{-2i\pi t\xi} (A_\Gamma(t) - P_{\text{on}}). \tag{24}$$

We decompose $A_\Gamma(t)$ into a sum over full cycles of the gene turning from ON to OFF, with the constraint that at time t the gene is ON:

$$A_\Gamma(t) = \sum_{k=0}^{\infty} A_{\Gamma k}(t), \tag{25}$$

where

$$A_{\Gamma k}(t) = x_{\text{on}}(t| \text{ ON at time 0 \& process has gone though k cycles}). \tag{26}$$

Since the first jump is from the ON to OFF, which is exponential it contributes $A_{\Gamma 0}(t) = e^{-k_{\text{off}} t}$.

First we compute an auxiliary probability distribution function of the time it takes the process to go through a full ON-OFF cycle $\eta(t)$ of taking an exponential jump out of the ON state followed by a Gamma distributed jump out of the OFF state:

$$\eta(t) = \int_0^t dx k_{\text{off}} e^{-k_{\text{off}} x} \frac{\beta^\alpha}{\Gamma(\alpha)} (t-x)^{\alpha-1} e^{-\beta(t-x)}. \tag{27}$$

The Fourier transform of this distribution is:

$$\hat{\eta}(\xi) = \int_0^{+\infty} dt e^{-2i\pi\xi t} \eta(t) = \frac{k_{\text{off}}}{2i\pi\xi + k_{\text{off}}} \frac{\beta^\alpha}{(2i\pi\xi + \beta)^\alpha}. \tag{28}$$

To compute $\hat{A}^*_\Gamma(\xi)$ we need to sum over all the possible times at which the cycles could have occurred, with the constraint that at time t the gene is ON:

$$\hat{A}^*_\Gamma(\xi) = \int_0^{+\infty} dt e^{-2i\pi\xi t} \left[\sum_{k=0}^{\infty} \left(\int_{t_i>0, \sum_{i=1}^k t_i < t} e^{-k_{\text{off}}(t - \sum_i t_i)} \prod_{i=1}^k \eta(t_i) dt_i \right) - P_{\text{on}} \right]. \tag{29}$$

We can rewrite the last term in Eq. 29:

$$\hat{A}^*_\Gamma(\xi) = \int_0^{+\infty} dt e^{-2i\pi\xi t} \left[\sum_{k=0}^{\infty} \left(\int_{t_i>0, \sum_{i=1}^k t_i < t} e^{-k_{\text{off}}(t - \sum_i t_i)} \prod_{i=1}^k \eta(t_i) dt_i \right) - P_{\text{on}} \sum_{k=0}^{\infty} \right.$$
$$\left. \int_{\sum_i t_i < t} (k_{\text{off}})^k e^{-k_{\text{off}} \sum_i t_i} e^{-k_{\text{off}}(t - \sum_i t_i)} \right], \tag{30}$$

using the expansion of unity:

$$1 = \sum_{k=0}^{\infty} e^{-k_{\text{off}} t} \frac{(k_{\text{off}} t)^k}{k!} \tag{31}$$
$$= \sum_{k=0}^{\infty} \int_{\sum_i t_i < t} (k_{\text{off}})^k e^{-k_{\text{off}} \sum_i t_i} e^{-k_{\text{off}}(t - \sum_i t_i)}, \tag{32}$$

with the convention for the $k = 0$ term:

$$\int_{\sum_i t_i < t} (k_{\text{off}})^k e^{-k_{\text{off}} \sum_i t_i} e^{-k_{\text{off}}(t - \sum_i t_i)} = e^{-k_{\text{off}} t}. \tag{33}$$

Collecting terms:

$$\sum_{k=0}^{\infty} \left[\int_{t_i>0} \prod_{i=1}^k dt_i \left[\left(\prod_{i=1}^k \eta(t_i) - P_{\text{on}} (k_{\text{off}})^k e^{-k_{\text{off}} \sum_i t_i} \right) \int_{t > \sum_i t_i} dt e^{-2i\pi\xi t} e^{-k_{\text{off}}(t - \sum_i t_i)} \right] \right] \tag{34}$$

and setting $u = t - \sum_i t_i$ in the last integral:

$$\hat{A}^*_\Gamma(\xi) = \sum_{k=0}^{\infty} \left[\int_{t_i>0} \prod_{i=1}^k dt_i \left[\left(\prod_{i=1}^k \eta(t_i) - P_{\text{on}} (k_{\text{off}})^k e^{-k_{\text{off}} \sum_i t_i} \right) \int_0^{+\infty} du e^{-2i\pi\xi(u + \sum_i t_i)} e^{-k_{\text{off}} u} \right] \right] \tag{35}$$

we obtain:

$$\hat{A}^*_\Gamma(\xi) = (k_{\text{off}} + 2i\pi\xi - k_{\text{off}}(1 + \frac{2i\pi\xi}{\beta})^{-\alpha})^{-1} - \frac{P_{\text{on}}}{2i\pi\xi}. \tag{36}$$

Using Eq. 21 we recover Eq. 11 in Materials and Methods of the main text. For $\alpha = 1$ we recover results of the two state model.

F. The average occupancy and autocorrelation of a Poisson-like polymerase firing model

We compared the autocorrelation function calculated for the two and three state cycle promoter models to the autocorrelation of the Poisson-like polymerase firing model. In this model we assume that the gene expression rate is memoryless and the transcription interval follows an exponential distribution with rate r:

$$P(t) = \frac{1}{\tau_P} e^{-rt}. \tag{37}$$

However, once a polymerase is loaded and starts transcribing, the gene must wait 6 seconds for the polymerase to leave the transcription initiation site before another polymerase can start transcribing. For this reason, the process is not a simple Poisson counting process, but includes a constant delay for every firing event. On average, there is a polymerase binding event every $T_{\text{eff}} = 1/r + 6$ s. T_{eff} is the effective time and $r_{\text{eff}} = 1/T_{\text{eff}}$ is the effective rate of this delayed Poisson process.

The quantity P_{on} corresponds to the average occupancy of polymerase binding sites over the duration of the cell cycle. The size of the polymerase is 150 bp and its speed is $\sim$ 25 bp/second, so the maximum loading rate of the polymerase is one every 6 second. Since the polymerase cannot load faster than once every 6 seconds, we calculate the average occupancy of the gene as the ratio of the average number of polymerase events within a given time window to the maximum number of polymerase events that could happen:

$$P_{\text{on}} = \frac{6}{T_{\text{eff}}} = \frac{6}{6 + 1/r}. \tag{38}$$

Note that, for the Poisson-like model, P_{on} does not correspond to an average time the gene spends in the ON state (since an OFF and ON state is not part of this model).

Using Eq. 38, we find that within the Poisson-like firing model, polymerase arrival rates are very heterogeneous across the embryo. In the anterior polymerases arrive at a rate $r = 1/6s^{-1}$, and in the boundary region $r = 1/54s^{-1}$.

Since the process is memoryless and the Poisson-like firing process is uncorrelated, its connected autocorrelation is close to a delta function $\delta(\tau = 0)$. However, due to the gene lengthy elongation time, there is a non-flat autocorrelation function of the fluorescence signal. At steady state, the connected autocorrelation function is:

$$\langle F(t)F(t+\tau)\rangle - \langle F(t)^2\rangle = P_{\text{on}} \sum_{i,j} L_i L_j A_P(j - \tau + i) - \left(P_{\text{on}} \sum_i L_i \right)^2, \tag{39}$$

where $A_P(j - \tau + i)$ is the probability of the polymerase to be at position i at time τ, given it was at position j at time 0 in the Poisson-like firing model.

If $\tau < 6s$ then the two positions on the gene, i and j, share the same polymerase with a probability proportional to $|6 - \tau|$, taking equally distributed polymerase positions. If $\tau > 6s$, $A_P(\tau)$ is given by the probability that there is a polymerase at the second site, which is independent of what happened at the first site. The two cases give:

$$A_P(\tau) = \frac{\theta(6 - |\tau|)}{6} \left[(6 - |\tau|) + P_{\text{on}}|\tau| \right] + \theta(|\tau| - 6) P_{\text{on}}, \tag{40}$$

where θ is the Heavyside function. This function is flat for $\tau > 6s$ and the first part of the right hand side of Eq. 40 has little effect on the autocorrelation function over a cell cycle (as cell cycle duration is much bigger than $6s$). For this reason we use a flat function as a very good approximation for A_P in our analysis.

From the form of Eqs. 39 and 40 and the flat approximation of A_P we see that P_{on} is only a normalizing constant and the shape of the function is completely determined by the loop function L_i, which is known. We can compare the expected autocorrelation function of a Poisson-like model to data and find that it agrees quite well.

G. Numerical simulations

To simulate the time evolution of MCP-GFP loci's intensity, we used the Gillespie algorithm [1, 2] to predict the time it takes for the gene to switch between the states, the active ON state and the inactive OFF states. In all models we assume that the time of the transition from the active to the inactive states, τ_{on} is exponentially distributed with rate k_{off}. The time of the transition from the inactive OFF states to ON state, τ_{off} depends on the model considered:

- for the two-state model τ_{off} is exponentially distributed with rate k_{on}.

- for the three-state model τ_{off} is a sum of two exponential processes with rates k_1 and k_2 that describe the transitions between the two OFF states.
- for the Gamma model τ_{off} is sampled from the $\Gamma(\alpha, \beta)$ distribution defined in Eq. 19.

To generate the traces of length T from N nuclei, we first simulate a long trajectory of length $N \times T$, denoted as $X(t)$. To account for the incompressibility of the polymerase, we divide the traces into 6s intervals, which is the time the polymerase needs to cover a region of the gene equal to its own lengths. We assume that at each 6s time point, if the gene is in the ON state, there is a transcription initiation event by a single RNA polymerase with a full transcription rate, defined as the length of the gene divided by the polymerase velocity, defined in SI section I A. Following this event, the RNA polymerase will slide along the target gene segment and synthesize a nascent RNA. At time i into this elongation process, the nascent RNA has L_i MS2 binding sites as depicted in Fig. 3 of the main text. To impose $P_{\text{on}} = k_{\text{on}}^{\text{eff}}/(k_{\text{on}}^{\text{eff}} + k_{\text{off}})$, if the gene switches into the OFF state before a full 6s interval, the polymerase transcribes the gene at a reduced rate proportional to the fraction of the 6s interval for which the gene was ON. The number of MS2 binding sites at the transcription locus site is therefore given by the convolution of the gene state and the promoter construct design function L (see Fig. 1 in the main text):

$$F(t) = X(t) * L. \tag{41}$$

We assume that the number of MCP-GFP molecules in the nuclei is sufficient to bind to all newly transcribed MS2 binding sites and that the binding process is infinitely fast. The spot intensity is calculated as the number of binding sites produced at the loci (given the intensity of each MPC-GFP dimer equal to 1). Lastly, the long spot intensity traces are divided equally into N smaller traces of length T.

H. Correction to the autocorrelation function for finite trace lengths

The short duration of the experimental traces, $v_{\alpha,i}$, where $1 \leq \alpha \leq M$ describes the identity of the trace and $0 < i < N$ denotes the sampling times, coupled with the need to correct for experimental biases by calculating the connected correlation function introduces finite size effects. The true connected correlation function between time points at a distance r, C_r (red line in Fig. 5), is not equal to the empirical connected correlation function calculated as an average over the M traces, $c(r)$ (blue line in Fig. 5), of the autocorrelation functions of the finite traces. The theoretical connected autocorrelation function calculated in our model is:

$$C_r = \frac{\langle v_i v_{i+r} \rangle - \bar{v}^2}{\bar{v^2} - \bar{v}^2}, \tag{42}$$

where $\langle \cdot \rangle$ denotes an average over random realizations of the process and we assume steady state $\bar{v^k} = \langle v_i^k \rangle = \langle v_{i+j}^k \rangle$. The empirical connected correlation function of each finite trace of length $N << \infty$ has the form:

$$c_\alpha(r) = \left[\frac{\sum\limits_{(i,j),|i-j|=r} \left\{ \left(v_{\alpha i} - \frac{1}{N}\sum\limits_{l=1}^{N} v_{\alpha l} \right) \left(v_{\alpha j} - \frac{1}{N}\sum\limits_{l=1}^{N} v_{\alpha l} \right) \right\}}{\frac{N-r}{N} \sum\limits_{j=1}^{N} \left(v_{\alpha j} - \frac{1}{N}\sum\limits_{l=1}^{N} v_{\alpha l} \right)^2} \right] \tag{43}$$

and the empirical connected correlation function calculated averaged over M traces is

$$c(r) = \frac{1}{M} \sum_{\alpha=1}^{M} c_\alpha(r). \tag{44}$$

C_r requires knowing the true second moment of the fluorescence signal $\bar{v^2}$. In our data we find that the true variance of the normalized fluorescence signal, $\bar{v^2} - \bar{v}^2$ is well approximated by the average over traces, so we approximate Eq. 43 by:

$$c_\alpha(r) = \frac{\sum\limits_{(i,j),|i-j|=r} \left\{ \left(v_{\alpha i} - \frac{1}{N}\sum\limits_{l=1}^{N} v_{\alpha l} \right) \left(v_{\alpha j} - \frac{1}{N}\sum\limits_{l=1}^{N} v_{\alpha l} \right) \right\}}{(N-r)(\bar{v^2} - \bar{v}^2)}. \tag{45}$$

The difference between the theoretical and empirical connected correlation function is independent of our model and arises for the connected correlation function of any random process, as shown in Fig. 5 for the simplest random process – the Ornstein-Uhlenbeck process. The difference is due to the fact that the short time average induces spurious correlations when calculating averages of the signal taken at different times. When analyzing the data, to avoid describing nucleus-to-nucleus variability that is not connected to the signal, we first subtract the mean steady state fluorescence signal of each trace, normalize this connected autocorrelation function to 1 at time $t = 0$, and then average over traces (Eq. 45) before averaging over the trace ensemble (Eq. 44). In steady state, the infinite trace mean equals the ensemble average, $\lim_{N\to\infty} \frac{1}{N}\sum_{i=1}^{N} v_{\alpha i} = \bar{v}$. However, as shown in Fig. 2 of the main text, the short trace mean is not a good approximation to the long term (or ensemble) average, $\frac{1}{N}\sum_{i=1}^{N} v_{\alpha i} \neq \bar{v}$. The points located in the center of the trace are much more correlated with the mean than the points at the beginning and end of the time interval. The correction for each value of r is different and must be separately computed.

In analyzing our data we use the finite size correction for the mean derived below that expresses the empirical connected correlation function $c(r)$ in terms of the theoretical connected correlation function C_r. For $N \to \infty$ the empirical connected correlation function becomes the infinite time connected correlation function, however our traces are very short. These corrections are valid for all time dependent data sets so for completeness the finite size correction for the variance is derived in SI Section II but is not used in the analysis.

The number of pairs of time points of distance r in a trace of length N is simply $N - r$ and the combination of Eqs. 44 and Eqs. 45 becomes:

$$\begin{aligned}
c(r) &= \frac{1}{M(N-r)(\bar{v^2}-\bar{v}^2)} \sum_{\alpha=1}^{M} \left[\sum_{i=1}^{N-r} \left\{ \left(v_{\alpha i} - \frac{1}{N}\sum_{l=1}^{N} v_{\alpha l} \right) \left(v_{\alpha(i+r)} - \frac{1}{N}\sum_{l=1}^{N} v_{\alpha l} \right) \right\} \right] \qquad (46)\\
&= \frac{1}{M(N-r)(\bar{v^2}-\bar{v}^2)} \sum_{\alpha=1}^{M} \Big[\sum_{i=1}^{N-r} \Big\{ v_{\alpha i} v_{\alpha(i+r)} - v_{\alpha i} \left(\frac{1}{N}\sum_{l=1}^{N} v_{\alpha l} \right) - \left(\frac{1}{N}\sum_{l=1}^{N} v_{\alpha l} \right) v_{\alpha(i+r)} + \\
&\quad (N-r) \left(\frac{1}{N}\sum_{l=1}^{N} v_{\alpha l} \right)^2 \Big\} \Big] \\
&= \left\langle \frac{1}{(\bar{v^2}-\bar{v}^2)} \left\{ \sum_{i=1}^{N-r} \frac{v_{\alpha i} v_{\alpha(i+r)}}{N-r} - \sum_{i=1}^{N-r} \frac{v_{\alpha i}}{N-r} \left(\frac{1}{N}\sum_{l=1}^{N} v_{\alpha l} \right) - \sum_{i=r+1}^{N} \frac{v_{\alpha i}}{N-r} \left(\frac{1}{N}\sum_{l=1}^{N} v_{\alpha l} \right) + \frac{1}{N^2} \left(\sum_{l=1}^{N} v_{\alpha l} \right)^2 \right\} \right\rangle_\alpha ,
\end{aligned}$$

where we have explicitly written out the terms and in the last line we introduced the average over traces $\langle \cdot \rangle_\alpha = 1/M \sum_{\alpha=1}^{M} \cdot$. In steady state due to time invariance:

$$\left\langle \sum_{i=N-r+1}^{N} \frac{v_{\alpha i}}{N-r} \left(\frac{1}{N}\sum_{l=1}^{N} v_{\alpha l} \right) \right\rangle_\alpha = \left\langle \sum_{i=1}^{r} \frac{v_{\alpha i}}{N-r} \left(\frac{1}{N}\sum_{l=1}^{N} v_{\alpha l} \right) \right\rangle_\alpha \qquad (47)$$

and the theoretical (not connected) correlation between two points is a function only of the distance between these two points:

$$\tilde{C}_r = \langle v_i v_{i+r} \rangle = 1/M \sum_{\alpha=1}^{M} v_{\alpha i} v_{\alpha i+r}. \qquad (48)$$

We have assumed that M is large and a population average over the M traces for points separated by r on each trace approximates the $M \to \infty$ limit of the theoretical average over different realizations of the process. Using Eq. 48 we obtain:

$$c(r) = \frac{\tilde{C}_r}{\bar{v^2}-\bar{v}^2} + \frac{1}{\bar{v^2}-\bar{v}^2} \left\langle \sum_{i=1}^{r} \frac{2v_{\alpha i}}{N-r} \left(\frac{1}{N}\sum_{l=1}^{N} v_{\alpha l} \right) + \frac{1}{N}\left(\frac{1}{N} - \frac{2}{(N-r)} \right) \left(\sum_{l=1}^{N} v_{\alpha l} \right)^2 \right\rangle_\alpha . \qquad (49)$$

To rewrite $\left\langle \left(\sum_{l=1}^{N} v_{\alpha l} \right)^2 \right\rangle_\alpha$ as a sum over $\tilde{C}_r$ we calculate the number of pairs of time points separated by a distance

k in the whole trace of length N. For $k = 0$ it is equal to N and for $1 \leq k \leq N-1$ it is equal to $2(N-k)$:

$$\left\langle \left(\sum_{l=1}^{N} v_{\alpha l} \right)^2 \right\rangle_{\alpha} = N\tilde{C}_0 + \sum_{k=1}^{N-1} 2(N-k)\tilde{C}_k. \quad (50)$$

Similarly

$$\left\langle \sum_{i=1}^{r} \sum_{l=1}^{N} v_{\alpha i} v_{\alpha l} \right\rangle_{\alpha} = \left\langle \left(\sum_{i=1}^{r} v_{\alpha i} \right)^2 \right\rangle_{\alpha} + \left\langle \sum_{i=1}^{r} \sum_{l=r+1}^{N} v_{\alpha i} v_{\alpha l} \right\rangle_{\alpha} \quad (51)$$

$$= r\tilde{C}_0 + \sum_{k=1}^{r-1} 2(r-k)\tilde{C}_k + \sum_{l=r+1}^{N} \sum_{i=1}^{r} \tilde{C}_{|l-i|} \quad (52)$$

$$= r\tilde{C}_0 + \sum_{k=1}^{r-1} 2(r-k)\tilde{C}_k + \sum_{m=1}^{N-1} \tilde{C}_m [\min(m+r, N) - \max(r, m)]. \quad (53)$$

Collecting the empirical connected autocorrelation function in Eq. 44 is expressed in terms of the theoretical non-connected correlation function in Eq 48 as:

$$c(r) = \frac{1}{\bar{v^2} - \bar{v}^2} \left[\tilde{C}_r + \frac{1}{N} \left(\frac{1}{N} - \frac{2}{(N-r)} \right) \left(N\tilde{C}_0 + \sum_{k=1}^{N-1} 2(N-k)\tilde{C}_k \right) + \frac{2}{N(N-r)} \left(r\tilde{C}_0 + \sum_{k=1}^{r-1} 2(r-k)\tilde{C}_k + \sum_{m=1}^{N-1} \tilde{C}_m [\min(m+r, N) - \max(r, m)] \right) \right]. \quad (54)$$

$\tilde{C}_k$ is the theoretical steady state non-connected correlation function of the process (given in Eq. 6 of the main text for the two state model, Eq. 8 of the main text for the cycle model and as the Fourier transform of Eq. 12 for the Γ model) and the average is over random realizations of the process. The mean and variance of the signal, $\bar{v}$ and $\bar{v^2}$, provide a normalization factor that is constant for all time differences r. We normalize the autocorrelation function setting the second term to 1 and these terms are not needed for the inference.

I. Correction to the autocorrelation function from correlations in the variance

In SI Section I H we calculated the finite size correction due to short traces for the empirical connected correlation function assuming that differences between the empirical variance and the theoretical variance for infinite traces do not affect the connected autocorrelation function. This approximation is valid for our data. For completeness we now calculate the finite size correction coming from spurious correlations in the variance obtained when computing the variance trace by trace, before averaging over the traces (Eq. 44). Analyzing the data, we normalize the autocorrelation function of each trace before taking the average over all traces because of potential nucleus-to-nucleus variability in the signal calibration. This is equivalent to dividing each autocorrelation function by its variance, before averaging over the traces and can introduce errors.

The empirical connected correlation function in Eqs. 44 and 43 can be rewritten by adding and subtracting 1 in the denominator as:

$$c(r) = \frac{N}{(N-r)(\bar{v^2} - \bar{v}^2)} \langle \left[\frac{\sum_{(i,j),|i-j|=r} \left\{ \left(v_{\alpha i} - \frac{1}{N} \sum_{l=1}^{N} v_{\alpha l} \right) \left(v_{\alpha j} - \frac{1}{N} \sum_{l=1}^{N} v_{\alpha l} \right) \right\}}{1 + \frac{1}{\bar{v^2} - \bar{v}^2} \left(\sum_{j=1}^{N} \left(v_{\alpha j} - \frac{1}{N} \sum_{l=1}^{N} v_{\alpha l} \right)^2 - (\bar{v^2} - \bar{v}^2) \right)} \right] \rangle_{\alpha},$$

where the average $\langle \cdot \rangle_{\alpha}$ is over M traces as defined in SI Section I H. Assuming the true variance of the process is close

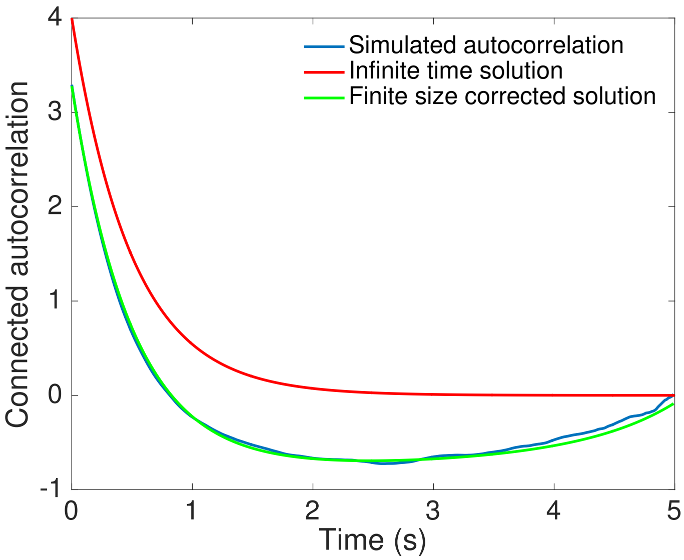

FIG. 5: **The finite trace effect for the Ornstein-Uhlenbeck process.** The connected autocorrelation function $C_r = \exp(-t/\tau)$ (red line) compared to the connected autocorrelation function calculated from short time traces as described in SI Section I H (blue line) and the corrected connected autocorrelation function (Eq. 54 green line). $\lambda = 2\mathrm{s}^{-1}$, $\gamma = 4\mathrm{s}^{-1/2}$ and the short trace length is 5s where the Ornstein-Ulhenbeck process is $\partial_t x = -\lambda x + \gamma \xi$ and ξ is Gaussian white noise.

to the empirical variance we linearize the denominator :

$$c(r) = \frac{N}{(N-r)(\bar{v^2} - \bar{v}^2)} \Big\langle \left[\sum_{(i,j),|i-j|=r} \left\{ \left(v_{\alpha i} - \frac{1}{N} \sum_{l=1}^{N} v_{\alpha l} \right) \left(v_{\alpha j} - \frac{1}{N} \sum_{l=1}^{N} v_{\alpha l} \right) \right\} \right] \times \left[2 - \frac{1}{\bar{v^2} - \bar{v}^2} \sum_{j=1}^{N} \left(v_{\alpha j} - \frac{1}{N} \sum_{l=1}^{N} v_{\alpha l} \right)^2 \right] \Big\rangle_\alpha . \quad (55)$$

The first term in the paranthesis is proportional to the connected correlation function in Eq. 54 we calculated in SI Section I H assuming constant variance. We focus on the second term:

$$\begin{aligned} d(r) &= \Big\langle \left[\sum_{(i,j),|i-j|=r} \left\{ \left(v_{\alpha i} - \frac{1}{N} \sum_{l=1}^{N} v_{\alpha l} \right) \left(v_{\alpha j} - \frac{1}{N} \sum_{l=1}^{N} v_{\alpha l} \right) \right\} \right] \cdot \left[\sum_{j=1}^{N} \left(v_{\alpha j} - \frac{1}{N} \sum_{l=1}^{N} v_{\alpha l} \right)^2 \right] \Big\rangle_\alpha \\ &= \Big\langle \left\{ \sum_{i=1}^{N-r} v_{\alpha i} v_{\alpha (i+r)} - \sum_{i=1}^{N-r} v_{\alpha i} \left(\frac{1}{N} \sum_{l=1}^{N} v_{\alpha l} \right) - \sum_{i=r+1}^{N} v_{\alpha i} \left(\frac{1}{N} \sum_{l=1}^{N} v_{\alpha l} \right) + \frac{N-r}{N^2} \left(\sum_{l=1}^{N} v_{\alpha l} \right)^2 \right\} \times \\ &\qquad \left[\sum_{j=1}^{N} v_{\alpha j}^2 - \frac{2}{N} \sum_{j=1}^{N} \sum_{l=1}^{N} v_{\alpha j} v_{\alpha l} + \frac{N}{N^2} \sum_{j=1}^{N} \sum_{l=1}^{N} v_{\alpha j} v_{\alpha l} \right] \Big\rangle_\alpha . \end{aligned}$$

Using time invariance at steady state (Eq. 47) in the first factor and simplifying the algebra in the second factor:

$$d(r) = \left\langle \left[\sum_{i=1}^{N-r} v_{\alpha i} v_{\alpha(i+r)} - 2 \sum_{i=1}^{N-r} v_{\alpha i} \left(\frac{1}{N} \sum_{l=1}^{N} v_{\alpha l} \right) + \frac{N-r}{N^2} \left(\sum_{l=1}^{N} v_{\alpha l} \right)^2 \right] \cdot \left[\sum_{j=1}^{N} v_{\alpha j}^2 - \frac{1}{N} \sum_{j=1}^{N} \sum_{l=1}^{N} v_{\alpha j} v_{\alpha l} \right] \right\rangle_\alpha$$

$$= \left\langle \sum_{j=1}^{N} \sum_{i=1}^{N-r} v_{\alpha j}^2 v_{\alpha i} v_{\alpha(i+r)} - \frac{1}{N} \sum_{i=1}^{N-r} \sum_{j,l=1}^{N} v_{\alpha j} v_{\alpha l} v_{\alpha i} v_{\alpha(i+r)} - \frac{2}{N} \sum_{i=1}^{N-r} \sum_{j,l=1}^{N} v_{\alpha i} v_{\alpha l} v_{\alpha j}^2 + \frac{2}{N^2} \sum_{i=1}^{N-r} \sum_{j,k,l=1}^{N} v_{\alpha i} v_{\alpha j} v_{\alpha k} v_{\alpha l} \right.$$

$$\left. + \frac{N-r}{N^2} \sum_{j,k,l=1}^{N} v_{\alpha j}^2 v_{\alpha k} v_{\alpha l} - \frac{N-r}{N^3} \sum_{j,k,l,m=1}^{N} v_{\alpha j} v_{\alpha k} v_{\alpha l} v_{\alpha m} \right\rangle_\alpha .$$

The final correction for correlation due to correlations in the variance coming from short time traces is easily evaluated in terms of four-points correlation function $F(s,t,u) = \overline{v_i v_{i+s} v_{i+s+t} v_{i+s+t+u}}$.

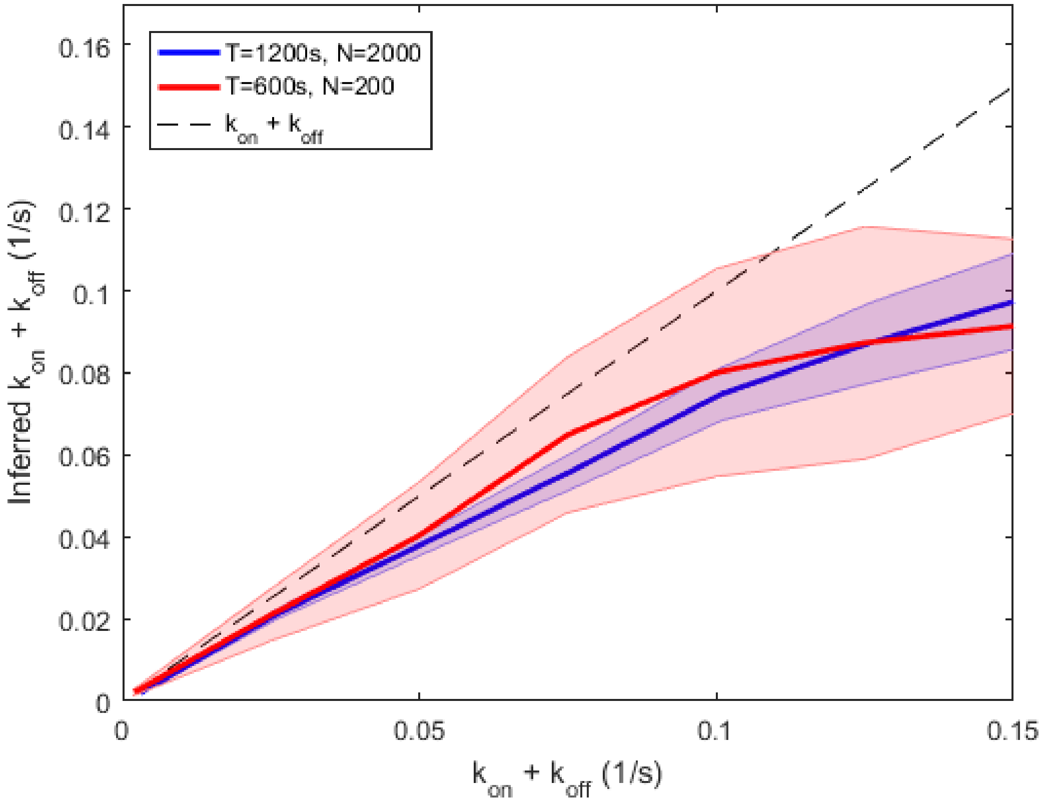

FIG. 6: **Inference of the two-state model from the cross-correlation function between $3'$ signals and $5'$ signals**. The gene cassette contains two identical arrays of MS2 binding sites on the $3'$ and $5'$ ends, separated by a gene of 3 kbp in length. The input parameters $k_{\rm on}$, $k_{\rm off}$ are varied so as to maintain the same $P_{\rm on} = 0.1$.

J. Cross-correlation

The presented correlation analysis can also be extended to constructs with two colored promoters inserted at two different positions on the same gene. In this case, each construct can have a different loop design function L_i^ν, where $\nu = 1, 2$, and the cross-correlation of the normalized fluorescence intensity is:

$$\langle F_1(t) F_2(s) \rangle = \sum_{i=1}^{r_1} \sum_{j=1}^{r_2} L_i^1 L_j^2 < a_i(t) a_j(s) > . \tag{56}$$

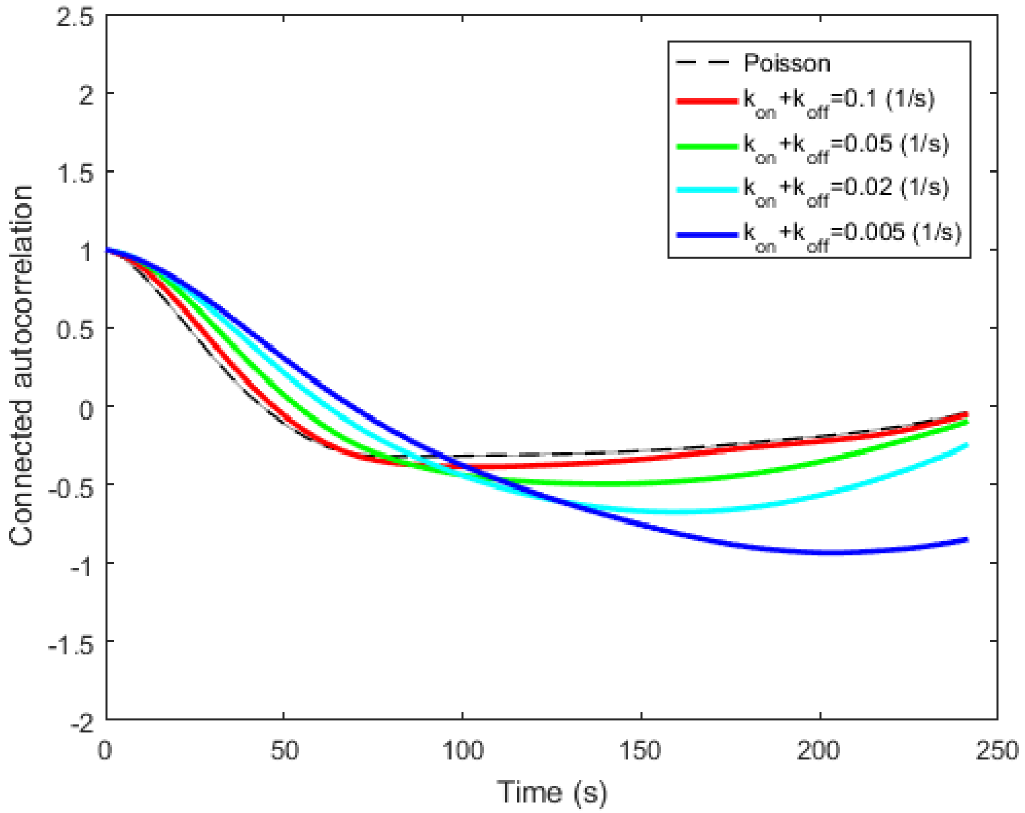

FIG. 7: **Comparison between the autocorrelation functions of the Poisson-like model and the two-state model**. Shown are the autocorrelation functions (calculated from 1000 traces of 250 s in length) of the Poisson-like model (dashed black) and the two-state model (solid) with varying k_{on} and k_{off}. The model parameters are set to achieve the same effective transcription rate, $P_{\mathrm{on}} = 0.1$, that we infer in the boundary region. For large $k_{on} + k_{off}$ values the shape of the autocorrelation function is dominated by the autocorrelation of the fluorescent probe and the Poisson-like and two state model autocorrelation functions look very similar. The inferred two state parameters are close to the green line. Since it is difficult to estimate the number of independent measurements, we cannot use standard statistical measures to compare these models with different numbers of parameters, whereas to determine the value of parameters within a given model we use a statistical measure (the mean square distance between the model prediction and data). For this reason we can differentiate between parameter values for the two state model that result in similar looking autocorrelation functions, but we cannot differentiate between two classes of models that result in similar differences in the autocorrelation functions.

The L_i^ν functions start at the same point (the one describing the downstream construct is 0 for the first steps).

After the loop design functions L_i^ν have been defined, the calculation of the theoretical cross-correlation function and autocorrelation rely only on calculating the correlations of the gene expression state, which is the same for both. So the results presented for the particular models are valid, after correcting for the two different loops functions. For examples, the steady state connected cross-correlation function of the two state model is:

$$\langle F_1(t)F_2(t+\tau)\rangle - \langle F_1(t)\rangle^2 = \sum_{i=1}^{r}\sum_{j=1}^{r} L_i L_j P_{\mathrm{on}} P_{\mathrm{off}} e^{|\tau - j + i|(\delta - 1)}, \tag{57}$$

where P_{on} and $\langle F_1(t)\rangle^2 = \langle F_2(t)\rangle^2$ can be independently calculated from either probe, which provides an independent estimate of the experimental noise.

The differences in the use of the cross-correlation function and autocorrelation function arise when calculating the finite size corrections from short traces, because assumptions about the statistical time invariance of the signal in steady state are no longer valid. The non-connected theoretical correlation function (equivalent of Eq. 48) is now defined on two signals, v_i and w_i:

$$\tilde{C}_r = \langle v_{\alpha,i} w_{\alpha,i+m}\rangle, \tag{58}$$

where $\langle\cdot\rangle$ define the average over random realizations of the process and in steady state is independent of i. Unlike for the autocorrelation function, $\tilde{C}_r$ is no longer symmetric with exchange of v_i and w_i. The empirical cross-correlation function is (assuming the variance is well approximated by the empirical variance):

$$c(r) = \left\langle \frac{1}{(\bar{v^2} - \bar{v}^2)} \left\{ \sum_{i=1}^{N-r} \frac{v_{\alpha i} w_{\alpha(i+r)}}{N-r} - \sum_{i=1}^{N-r} \frac{v_{\alpha i}}{N-r} \left(\frac{1}{N} \sum_{l=1}^{N} w_{\alpha l} \right) - \sum_{i=r+1}^{N} \frac{w_{\alpha i}}{N-r} \left(\frac{1}{N} \sum_{l=1}^{N} v_{\alpha l} \right) + \frac{1}{N^2} \left(\sum_{l=1}^{N} v_{\alpha l} \right) \left(\sum_{l=1}^{N} w_{\alpha l} \right) \right\} \right\rangle_{\alpha}, \quad (59)$$

which in terms of the $\tilde{C}_m$ is:

$$c(r) = \frac{1}{(\bar{v^2} - \bar{v}^2)} \left\{ \tilde{C}_r - \frac{1}{N(N-r)} \sum_{i=1}^{N-r} \sum_{l=1}^{N} \tilde{C}_{l-i} - \frac{1}{N(N-r)} \sum_{i=r+1}^{N} \sum_{l=1}^{N} \tilde{C}_{i-l} + \frac{1}{N^2} \sum_{i,l=1}^{N} \tilde{C}_{i-l} \right\}. \quad (60)$$

Repeating the steps in SI Section I H we obtain the finite size correction for the cross-correlation function.

$$\begin{aligned} c(r) = & \frac{1}{(\bar{v^2} - \bar{v}^2)} \left\{ \tilde{C}_r - \frac{1}{N(N-r)} \sum_{k=-N+r+1}^{N-r-1} (N-r-|k|)\tilde{C}_k \right. \\ & - \frac{1}{N(N-r)} \sum_{i=1}^{N-r} \sum_{l=N-r+1}^{N} \tilde{C}_{l-i} - \frac{1}{N(N-r)} \sum_{k=-N+r+1}^{N-r-1} (N-r-|k|)\tilde{C}_k \\ & \left. - \frac{1}{N(N-r)} \sum_{i=r+1}^{N} \sum_{l=1}^{r} \tilde{C}_{i-l} + \frac{1}{N^2} \sum_{k=-N+1}^{N-1} (N-|k|)\tilde{C}_k \right\} \\ = & \frac{1}{(\bar{v^2} - \bar{v}^2)} \left\{ \tilde{C}_r - \frac{2}{N(N-r)} \sum_{k=-N+r+1}^{N-r-1} (N-r-|k|)\tilde{C}_k - \frac{1}{N(N-r)} \sum_{m=1}^{N-1} \tilde{C}_m [\min(m+N-r, N) - \max(N-r, m)] \right. \\ & \left. - \frac{1}{N(N-r)} \sum_{m=1}^{N-1} \tilde{C}_m [\min(m+r, N) - \max(r, m)] + \frac{1}{N^2} \sum_{k=-N+1}^{N-1} (N-|k|)\tilde{C}_k \right\}. \end{aligned}$$

K. Precision of the translational process

The precision of the total mRNA produced during a cell cycle presented in the main text is proportional to the activity of the gene and requires a careful calculation of the variability of the probability of the gene to be ON in different nuclei at the same position. The total activity of a nucleus, defined as the integral of the normalized fluoresce $\sum_i^K F_i$, where $i < K$ are the sampling times in steady state window of the cycle, in steady state is proportional to the probability of the gene to be ON in a given trace, $P_{\rm on}^{\alpha}$. To keep our analysis independent of normalization, we will calculate the relative error defined as the variance over the mean of $P_{\rm on}^{\alpha}$, $\text{var}(P_{\rm on}^{\alpha})/\langle P_{\rm on}^{\alpha}\rangle_{\alpha}$, where the averages are taken over traces.

First, we can calculate the relative error of the probability of the gene to be ON $P_{\rm on}^{\alpha}$ directly from the traces. We compute the mean and standard deviation of the distribution of $P_{\rm on}^{\alpha}$ in a given window along the AP axis. $P_{\rm on}^{\alpha}$ for each trace is calculated from Eq. 4.

We can compare the results of the empirically estimated relative error to predictions of the steady state models. We know that the expected average over traces $\sum_{\alpha=1}^{M} P_{\rm on}^{\alpha}$ is $P_{\rm on}$. Within the assumption of our model presented in SI Section I B, the expectation value of the square of the $P_{\rm on}^{\alpha}$ is expressed in terms of the expression states of the gene, $X(t)$:

$$\langle P_{\rm on}^{\alpha,2} \rangle_{\alpha} = \left\langle \frac{1}{T^2} \int_0^T dt \int_0^T ds X(t) X(s) \right\rangle_{\alpha}, \quad (61)$$

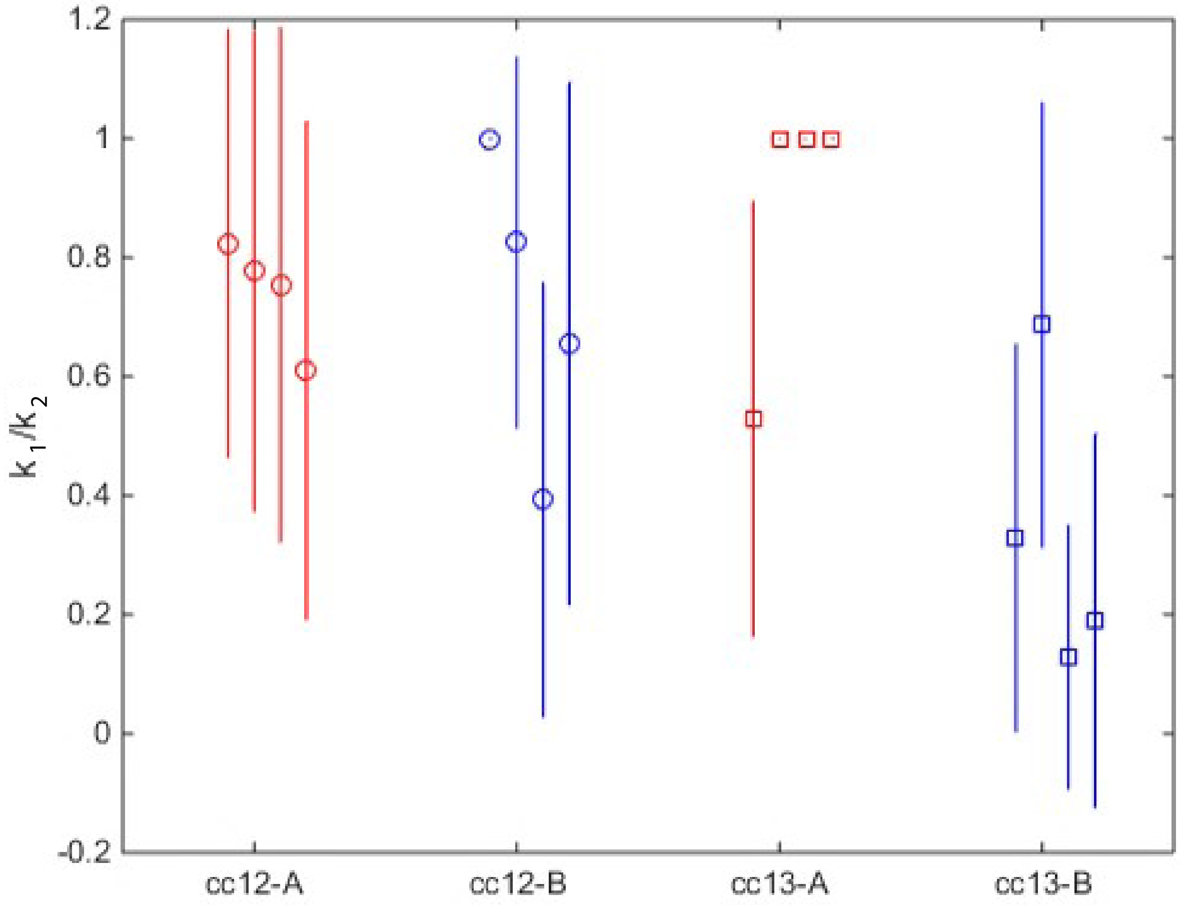

FIG. 8: **The fit of the three state cycle model to the data.** The fit of the ratio of the two rates for leaving the two OFF states, k_1/k_2, to the steady state traces from four embryos in the anterior and boundary region of cell cycle 12 and 13. Each point is data from one embryo. The error bars represent the standard deviation of the inferred value. The fit is for a randomized 60% of the data. The sum of the switching rates $k_{\rm on} + k_1 + k_2$ is shown in Fig. 5B of the main text.

where the average is over M traces and T is the total duration of the trace in real time. In terms of the probability that the gene is ON at time τ given that it was ON at time 0, $A(\tau)$ defined in Eq. 6, we obtain

$$\langle P_{\rm on}^{\alpha,2}\rangle_\alpha = \frac{1}{T^2}\int_0^T dt \int_0^T ds P_{\rm on} A(t-s), \tag{62}$$

where $A(\tau)$ has units of seconds. The relative error is obtained by replacing $A(\tau)$ by the appropriate function for each model. For the two state model:

$$\langle P_{\rm on}^{\alpha,2}\rangle_\alpha = \frac{P_{\rm on}}{T^2}\int_0^T dt \int_0^T ds (P_{\rm on} + P_{\rm off} e^{-|t-s|(k_{\rm on}+k_{\rm off})}). \tag{63}$$

Integrating and substracting the mean squared we obtain the relative error:

$$\frac{\delta P_{\rm on}}{P_{\rm on}} = \frac{1}{T}\sqrt{2\frac{k_{\rm off}}{k_{\rm on}(k_{\rm on}+k_{\rm off})}(T - \frac{1-e^{-T(k_{\rm on}+k_{\rm off})}}{k_{\rm on}+k_{\rm off}})}. \tag{64}$$

The probability of the gene to be on is proportional to the total mRNA produced and for large T we reproduce the result in Eq. 4 in the main text:

$$\frac{\delta \text{mRNA}}{\text{mRNA}} = \sqrt{\frac{2}{T}\frac{k_{\rm off}}{k_{\rm on}(k_{\rm on}+k_{\rm off})}} = \sqrt{2\frac{\tau_i(1-P_{\rm on})}{T P_{\rm on}}}. \tag{65}$$

For the three state cycle model the same calculation is valid until Eq. 62 and is then carried out numerically.

In the Poisson-like firing model the accuracy of *hunchback* mRNA production over one cell-cycle the average number of events within a cell-cycle of duration T is $n = T/T_{\rm eff} = rT/(6r+1)$. The amount of mRNA produced during the cell-cycle is proportional to the number of polymerase arrival events. Polymerase arrivals are Poisson-distributed and followed by a deterministic delay of $6s$ when the polymerase binding site is still occupied by the previous polymerase, so we find that the squared relative error is proportional to the inverse of the number of events times the error on the effective arrival rate of polymerases:

$$\frac{\langle mRNA^2\rangle - \langle mRNA\rangle^2}{\langle mRNA\rangle^2} = \frac{1}{n}\frac{\langle T_{\rm eff}^2\rangle - \langle T_{\rm eff}\rangle^2}{T_{\rm eff}^2} = \frac{6r+1}{rT}\frac{1/r}{(1/r+6)^2} = \frac{1}{T(6r+1)}. \quad (66)$$

Replacing with the expression of Eq. 38

$$\frac{\langle mRNA^2\rangle - \langle mRNA\rangle^2}{\langle mRNA\rangle^2} = \frac{1}{T}\frac{1}{1+P_{\rm on}/(1-P_{\rm on})} = \frac{6(1-P_{\rm on})}{T}, \quad (67)$$

and the relative error in the produced mRNA is

$$\frac{\delta \text{mRNA}}{\text{mRNA}} = \sqrt{\frac{6(1-P_{\rm on})}{T}}. \quad (68)$$

The predicted accuracy for both the boundary and anterior regions in the embryo is much higher than the experimentally observed accuracy.

Precision from static (Fluorescent In Situ Hybridization – FISH) images is calculated as the standard deviation over the mean of the distribution of a binary variable, which for each nucleus is 1 if the gene is on in the static image and 0 if it off [3–5]. The signal in FISH datasets in an average over an unknown timeframe. To compare our analysis of the time dependent signal to these previous measurements, we use a binary variable, which is 1 for each nucleus that was ON during the steady state interphase and 0 for each nucleus that was always OFF. The results of the relative error as a function of position obtained using this empirical analysis in SIFig. 9 show agreement with previous reports [5]: for most traces the relative error in the anterior is zero – all nuclei in a given AP axis window express, and it increases to $\sim 50\%$ at the boundary.

---

[1] D. T. Gillesple, **93555**, 2340 (1977).
[2] D. Bratsun, D. Volfson, L. S. Tsimring, and J. Hasty, PNAS **102**, 14593 (2005).
[3] T. Gregor, E. F. Wieschaus, A. P. McGregor, W. Bialek, and D. W. Tank, Cell **130**, 141 (2007), ISSN 0092-8674.
[4] A. Porcher and N. Dostatni, Current Biology **20**, R249 (2010), ISSN 1879-0445.
[5] S. C. Little, M. Tikhonov, and T. Gregor, Cell **154**, 789 (2013), ISSN 1097-4172.

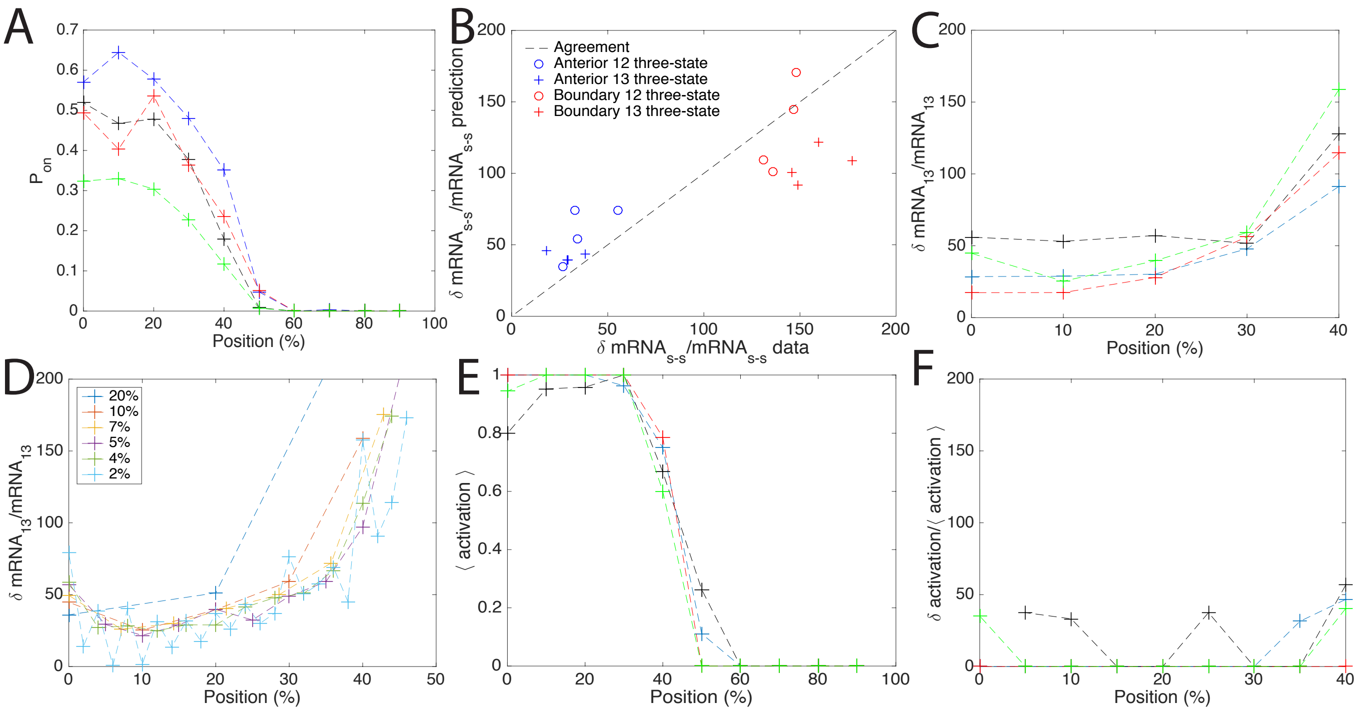

FIG. 9: **The relative error of gene expression.** A. The mean probability of the gene to be ON at any time during the cell cycle as a function of the embryo length (binary approximation). B. Comparison of the relative error in the mRNA produced during the steady state of the interphase estimated empirically from data (abscissa) and from theoretical arguments in Eq. 62 using the inferred parameters from the autocorrelation function (ordinate), in the anterior (blue) and the boundary (red) regions, show very good agreement. C. The conclusions about precision do not depend on the embryo. The relative error of the total mRNA produced in cell cycle 13 as a function of position for windows equal to 10% of the embyo length. Each colored line represents one embryo. The same data plotted as an average over embryos with the variance as error bars is shown in Fig. 7 of the main text. D. The conclusions about precision do not depend on the window size. The total mRNA produced in cell cycle 13 as a function of position for different window sizes. Except for very large scales (20%) and very small scales comparable to one nuclear width (2%, the relative error as a function of position is reproducible. E.The mean probability for the gene to be ON averaged over the cell cycle. F. The relative error of the discrete variable that describes the probability of the gene to be ON at any time during the cell cycle as function of position. The relative error is much lower in the anterior compared to the error in the total produced mRNA, but remains high at the boundary. In A, C, E and F each colored lines describe different embryos.

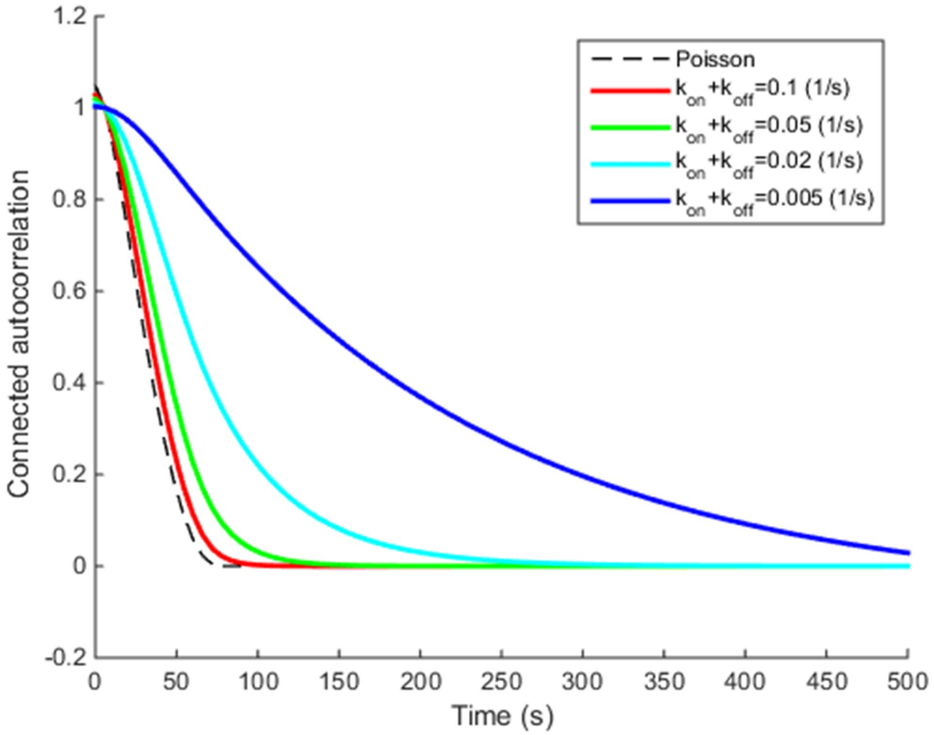

FIG. 10: **The autocorrelation function for Poisson-like model and the two-state model for infinitely-long time traces**. Autocorrelation functions of the Poisson-like model (dashed black) and the two-state models (solid) with $P_{\text{on}} = 0.1$ (similar to the inferred value in the boundary region) and varying $k_{on} + k_{off}$. In the inferred parameter regime (approximately green line), longer time traces do not help distinguish the two models based on the autocorrelation function. For large $k_{on} + k_{off}$ values the shape of the autocorrelation function is dominated by the autocorrelation of the fluorescent probe and the Poisson-like and two state model autocorrelation functions look very similar, even for long traces.)

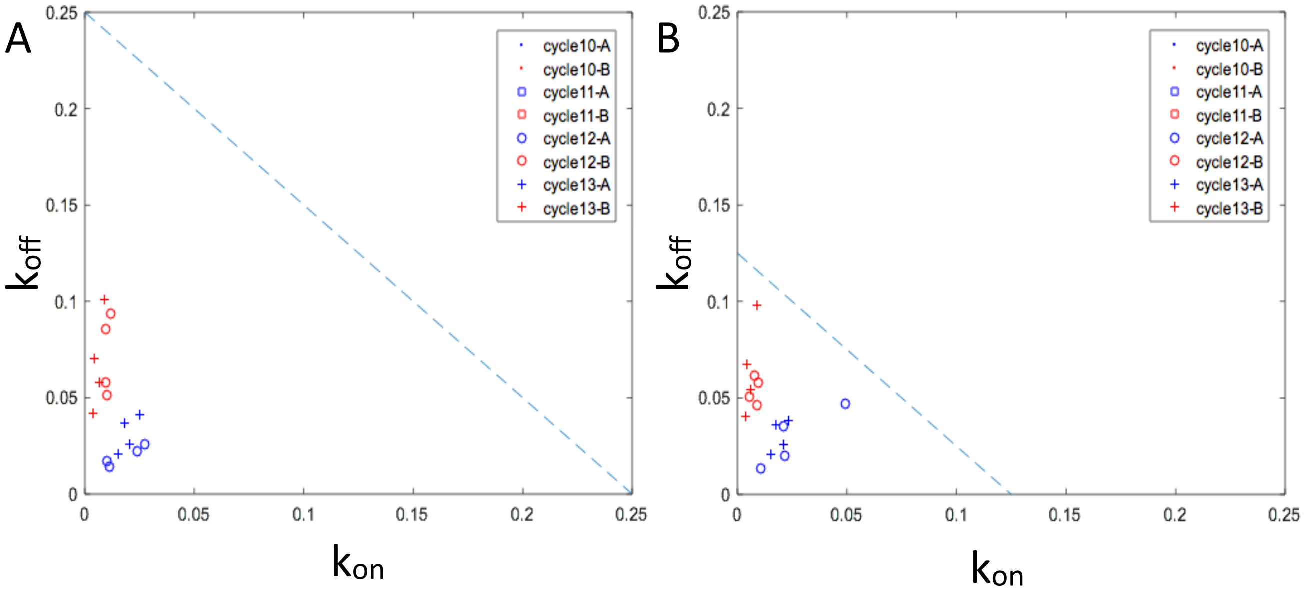

FIG. 11: **The dependence of the data fit on polymerase buffering time.** Assuming different buffering times for the polymerase does not strongly affect the fit of the switching rates: a fit with $\tau_{\text{buffering}} = 4s$ (A) and $\tau_{\text{buffering}} = 8s$. $\tau_{\text{buffering}} = 6s$ is used in the main text in Fig. 5D.
