# Supplementary figures and images for: Precision of Readout at the hunchback Gene: Analyzing Short Transcription Time Traces in Living Fly Embryos
Source: PLoS Comput Biol. 2016 Dec 12;12(12):e1005256. doi: 10.1371/journal.pcbi.1005256 (PMC5152799; doi:10.1371/journal.pcbi.1005256)

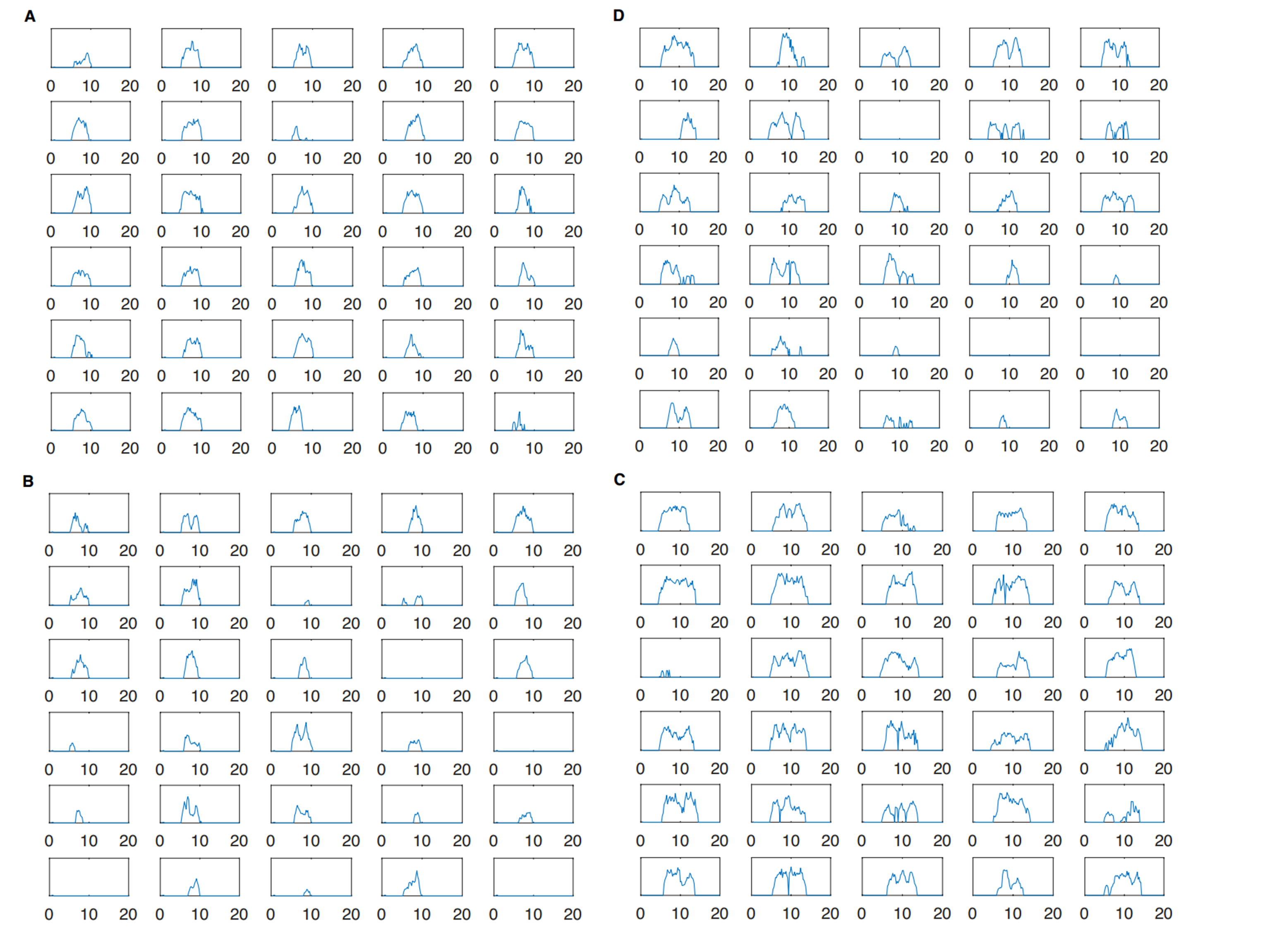

Supplement: S1 Fig — Consecutively shown are the traces in (A) Cycle 12, Anterior, (B) Cycle 12, Boundary (C) Cycle 13, Anterior, (D) Cycle 13, Boundary. The x axis is time in minute and y axis is the spot intensity in AU. (TIF) [file pcbi.1005256.s002.tif]

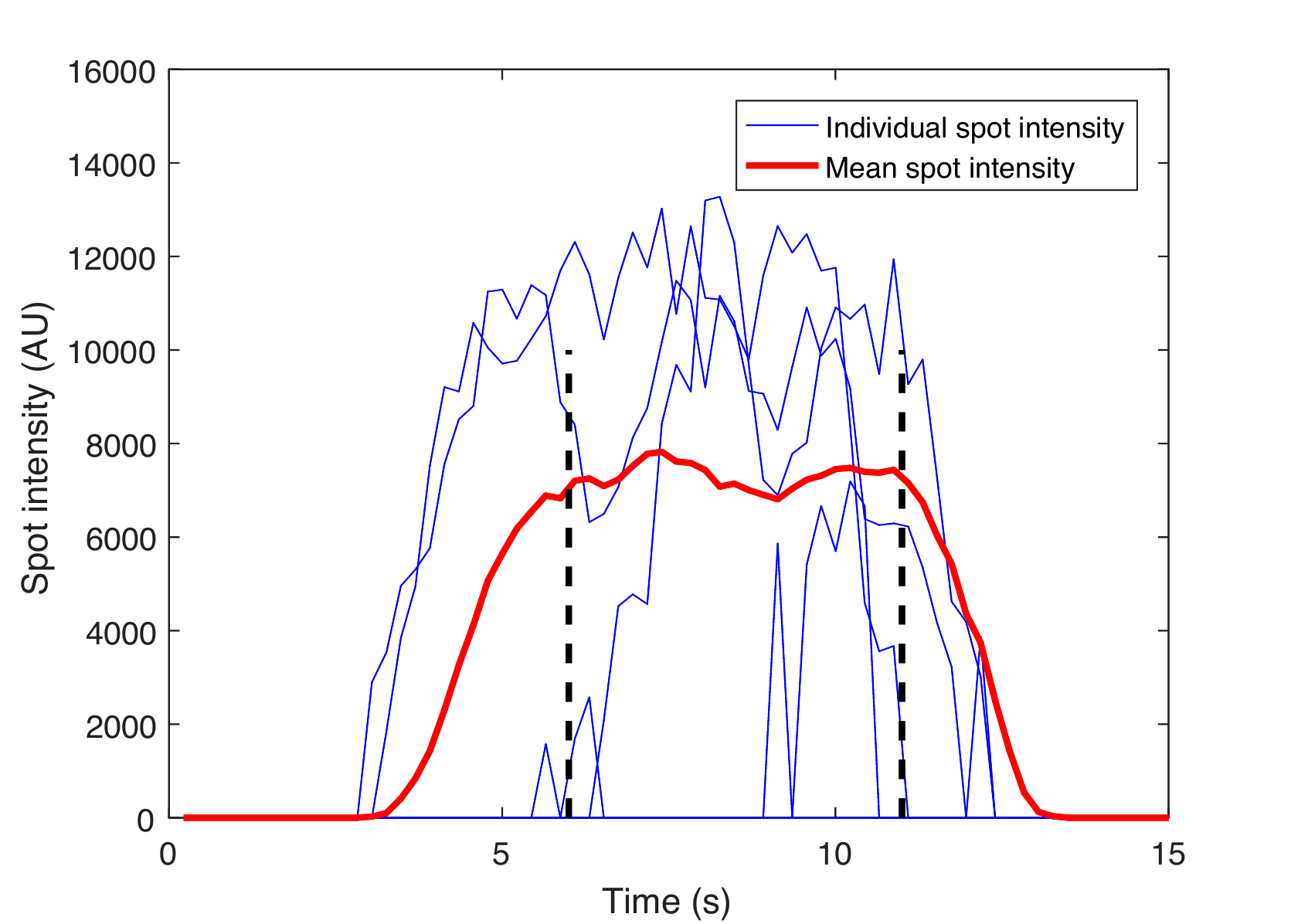

Supplement: S2 Fig — Shown are examples of 5 (out of 154) individual traces (blue) taken from embryo 1, cycle 13. Also shown is the mean spot intensity over time of all traces (red). The steady state window is chosen to be from the 6th minute to the 11th minute (dashed lines). (TIF) [file pcbi.1005256.s003.tif]

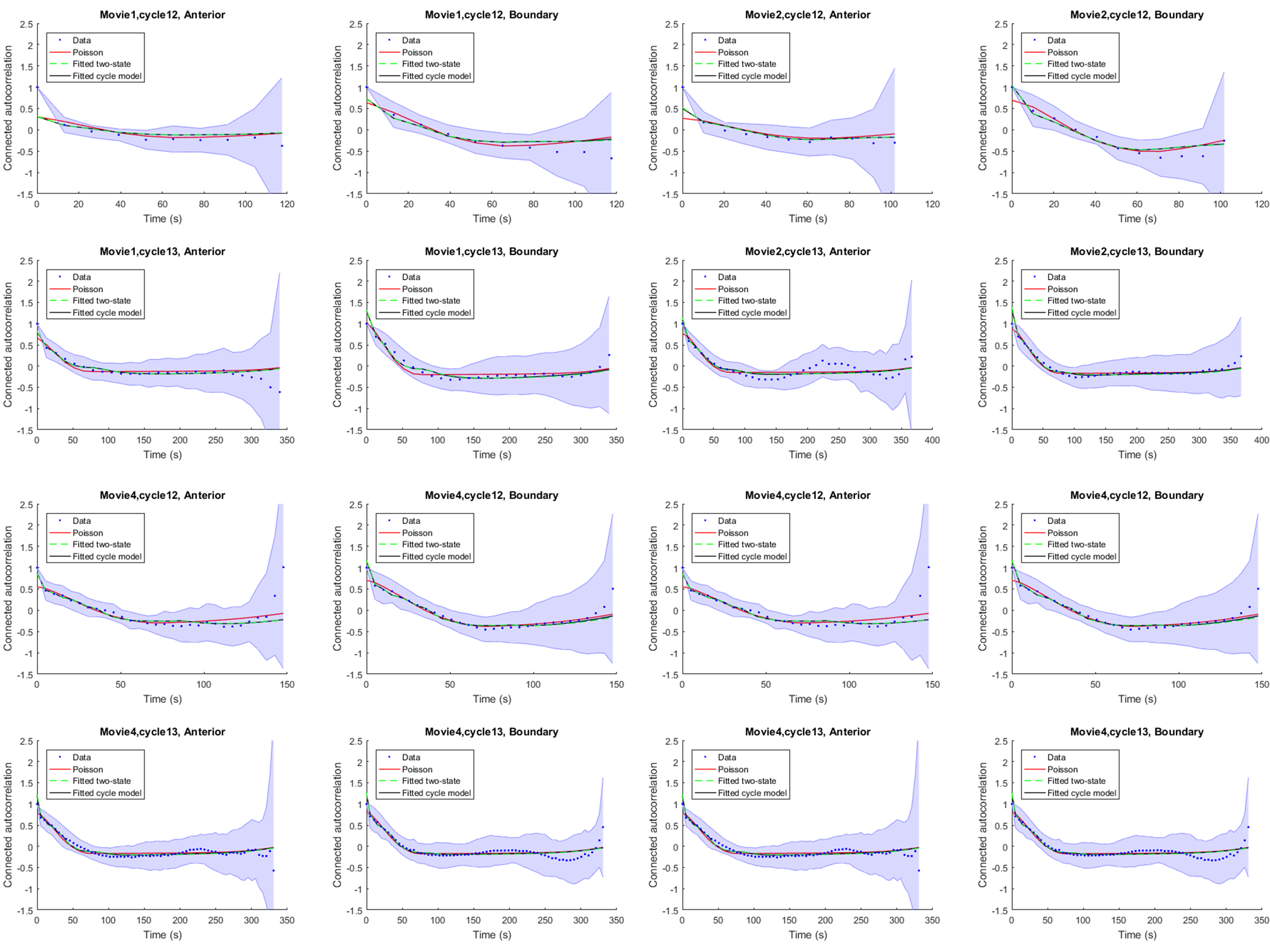

Supplement: S3 Fig — The empirical autocorrelation function (blue dots) for both the anterior and boundary regions in all four embryos is fit using the autocorrelation function with the finite size corrections for the Poisson-like model (red lines), two-state model (green lines) and three-state cycle model (black lines). (TIF) [file pcbi.1005256.s004.tif]

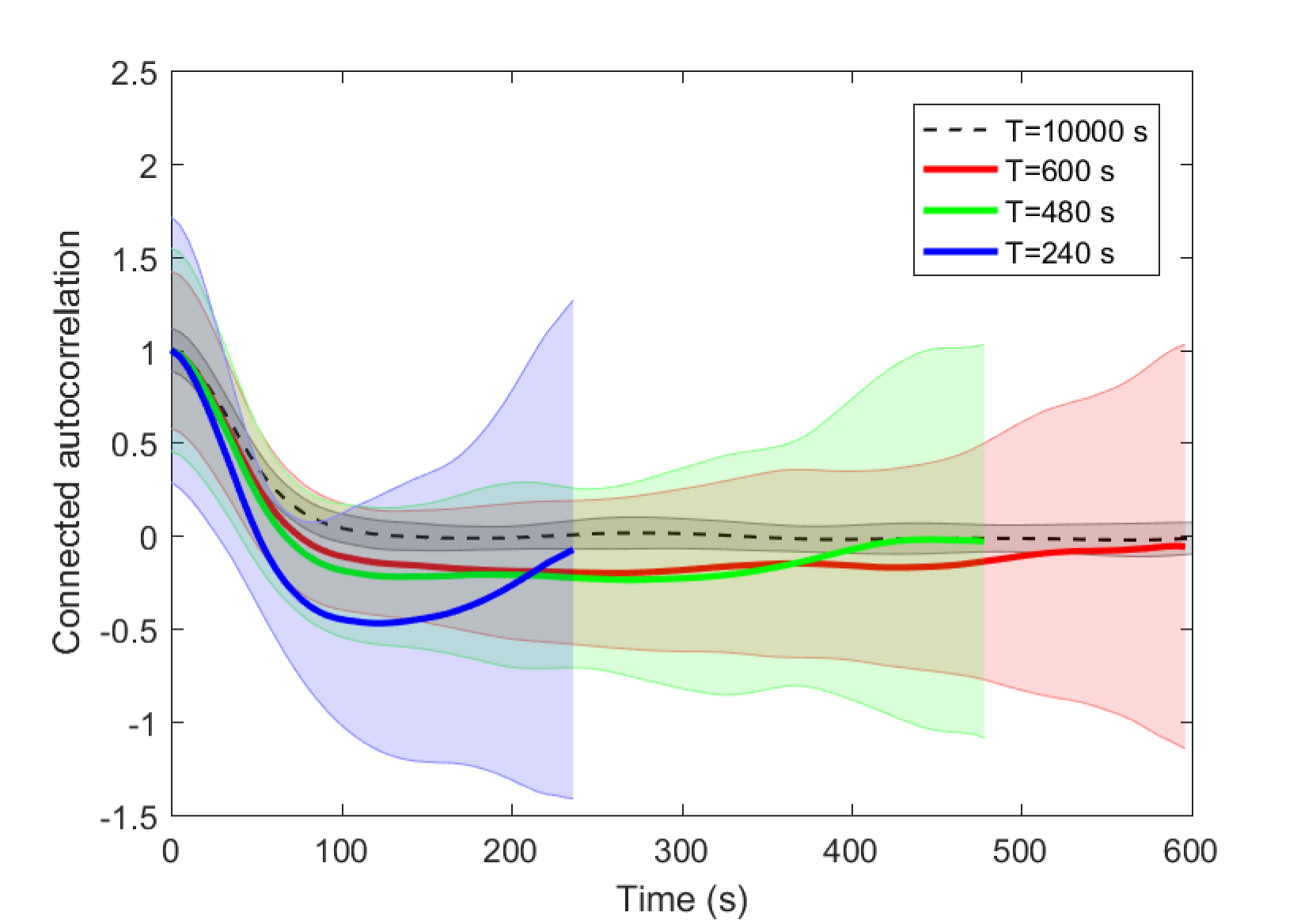

Supplement: S4 Fig — The shaded areas denote the standard variation over 500 simulated traces. The switching rates kon = koff = 0.01s−1. (TIF) [file pcbi.1005256.s005.tif]

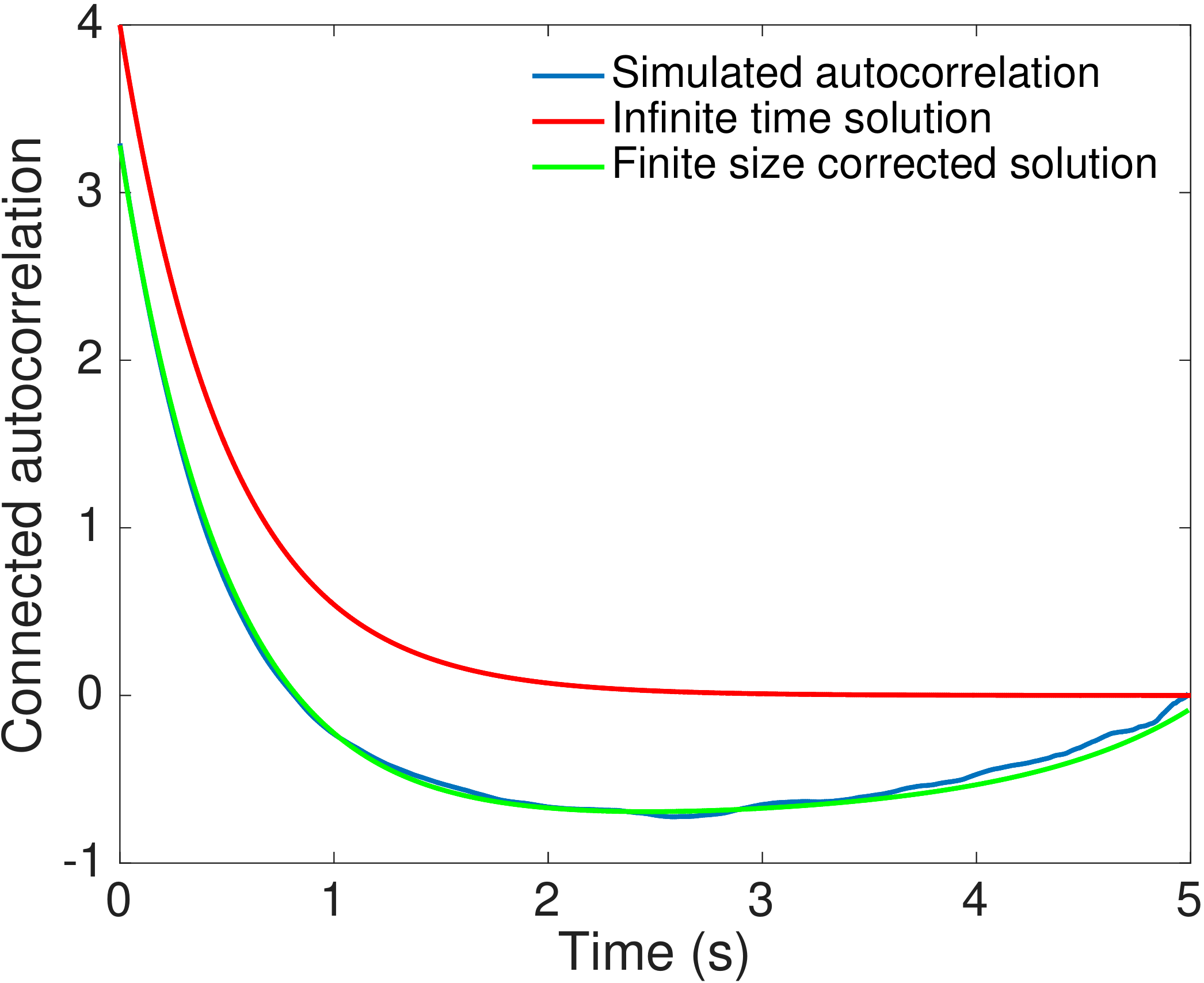

Supplement: S5 Fig — The connected autocorrelation function Cr = exp(−t/τ) (red line) compared to the connected autocorrelation function calculated from short time traces as described in SI Section I H (blue line) and the corrected connected autocorrelation function (Eq. 54). λ = 2s−1, γ = 4s−1/2 and the short trace length is 5s where the Ornstein-Ulhenbeck process is ∂t x = −λx + γξ and ξ is Gaussian white noise. (TIF) [file pcbi.1005256.s006.tif]

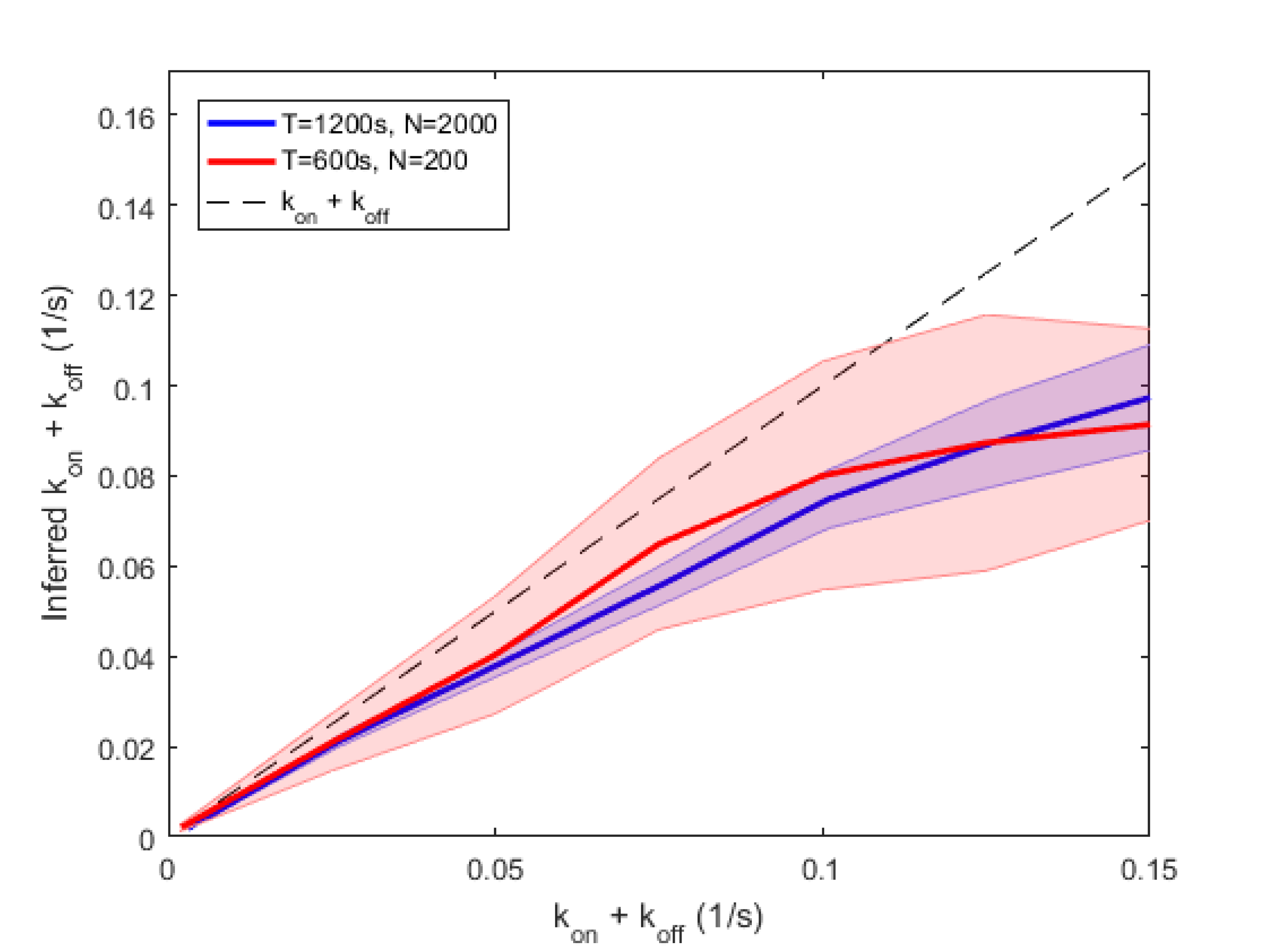

Supplement: S6 Fig — The gene cassette contains two identical arrays of MS2 binding sites on the 3′ and 5′ ends, separated by a gene of 3 kbp in length. The input parameters kon, koff are varied so as to maintain the same Pon = 0.1. (TIF) [file pcbi.1005256.s007.tif]

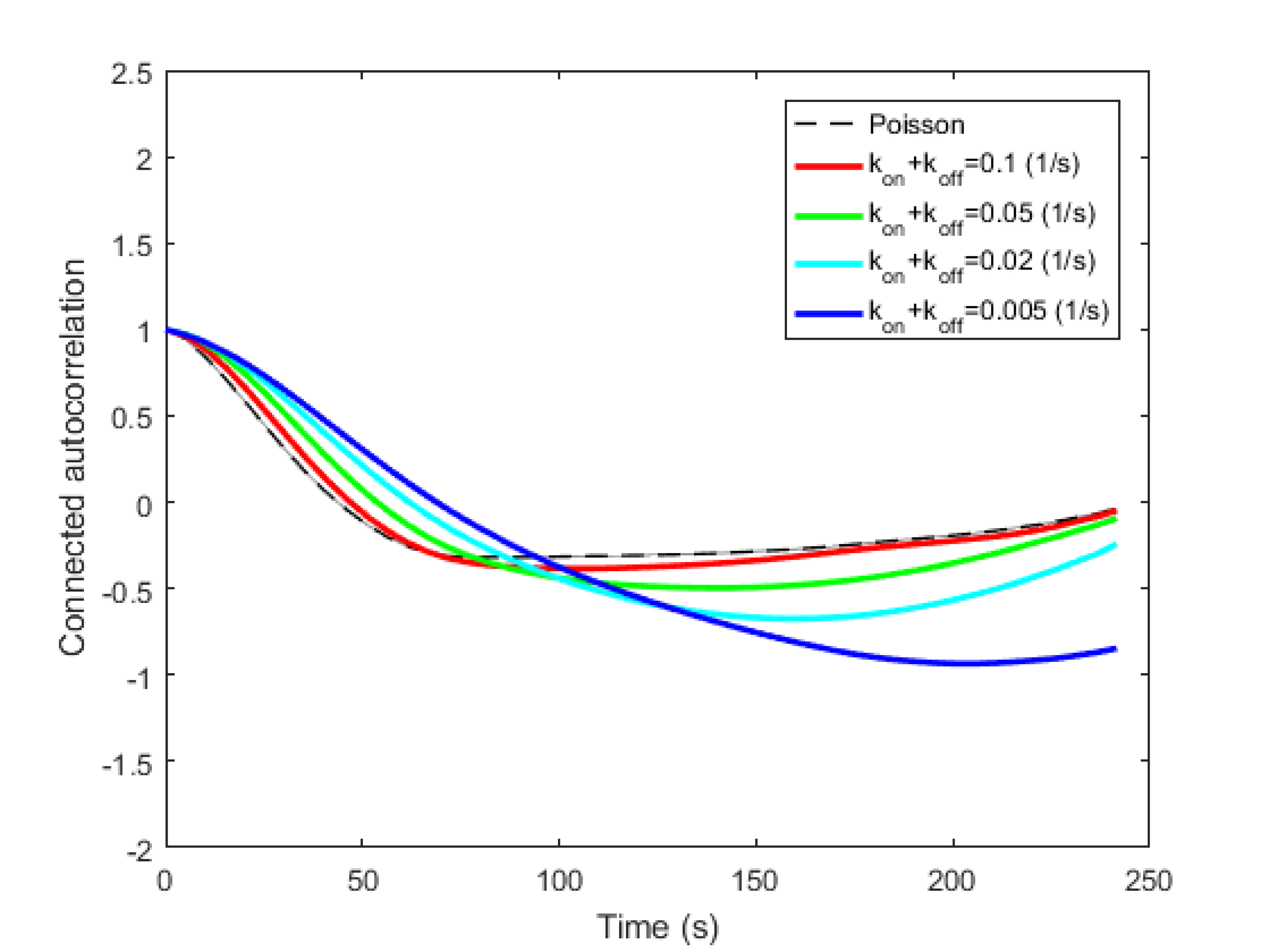

Supplement: S7 Fig — Shown are the autocorrelation functions (calculated from 1000 traces of 250 s in length) of the Poisson-like model (dashed black) and the two-state model (solid) with varying kon and koff. The model parameters are set to achieve the same effective transcription rate, Pon = 0.1, that we infer in the boundary region. For large kon + koff values the shape of the autocorrelation function is dominated by the autocorrelation of the fluorescent probe and the Poisson-like and two state model autocorrelation functions look very similar. The inferred two state parameters are close to the green line. Since it is difficult to estimate the number of independent measurements, we cannot use standard statistical measures to compare these models with different numbers of parameters, whereas to determine the value of parameters within a given model we use a statistical measure (the mean square distance between the model prediction and data). For this reason we can differentiate between parameter values for the two state model that result in similar looking autocorrelation functions, but we cannot differentiate between two classes of models that result in similar differences in the autocorrelation functions. (TIF) [file pcbi.1005256.s008.tif]

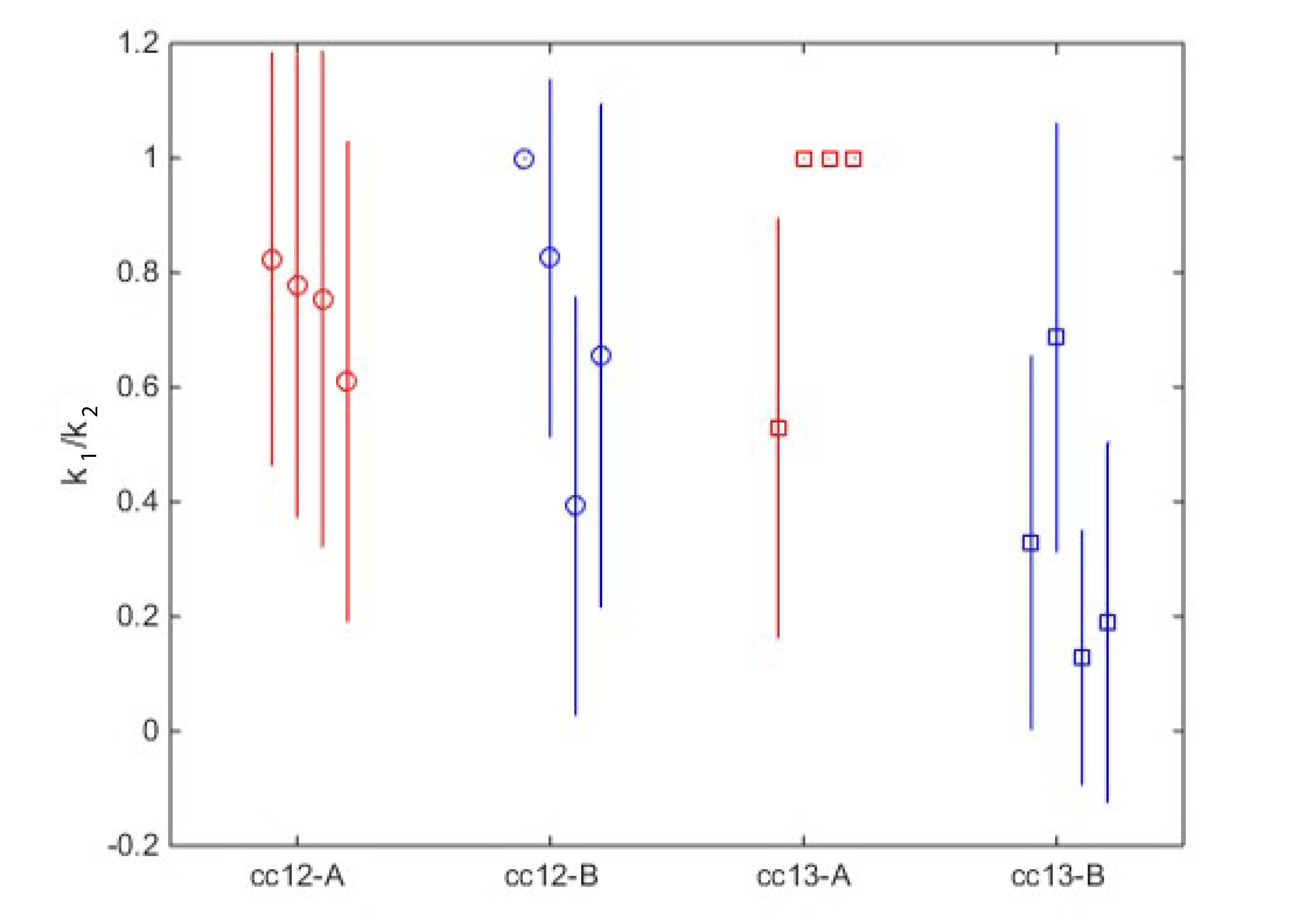

Supplement: S8 Fig — The fit of the ratio of the two rates for leaving the two OFF states, k1/k2, to the steady state traces from four embryos in the anterior and boundary region of cell cycle 12 and 13. Each point is data from one embryo. The error bars represent the standard deviation of the inferred value. The fit is for a randomized 60% of the data. The sum of the switching rates kon + k1 + k2 is shown in Fig 5B of the main text. (TIF) [file pcbi.1005256.s009.tif]

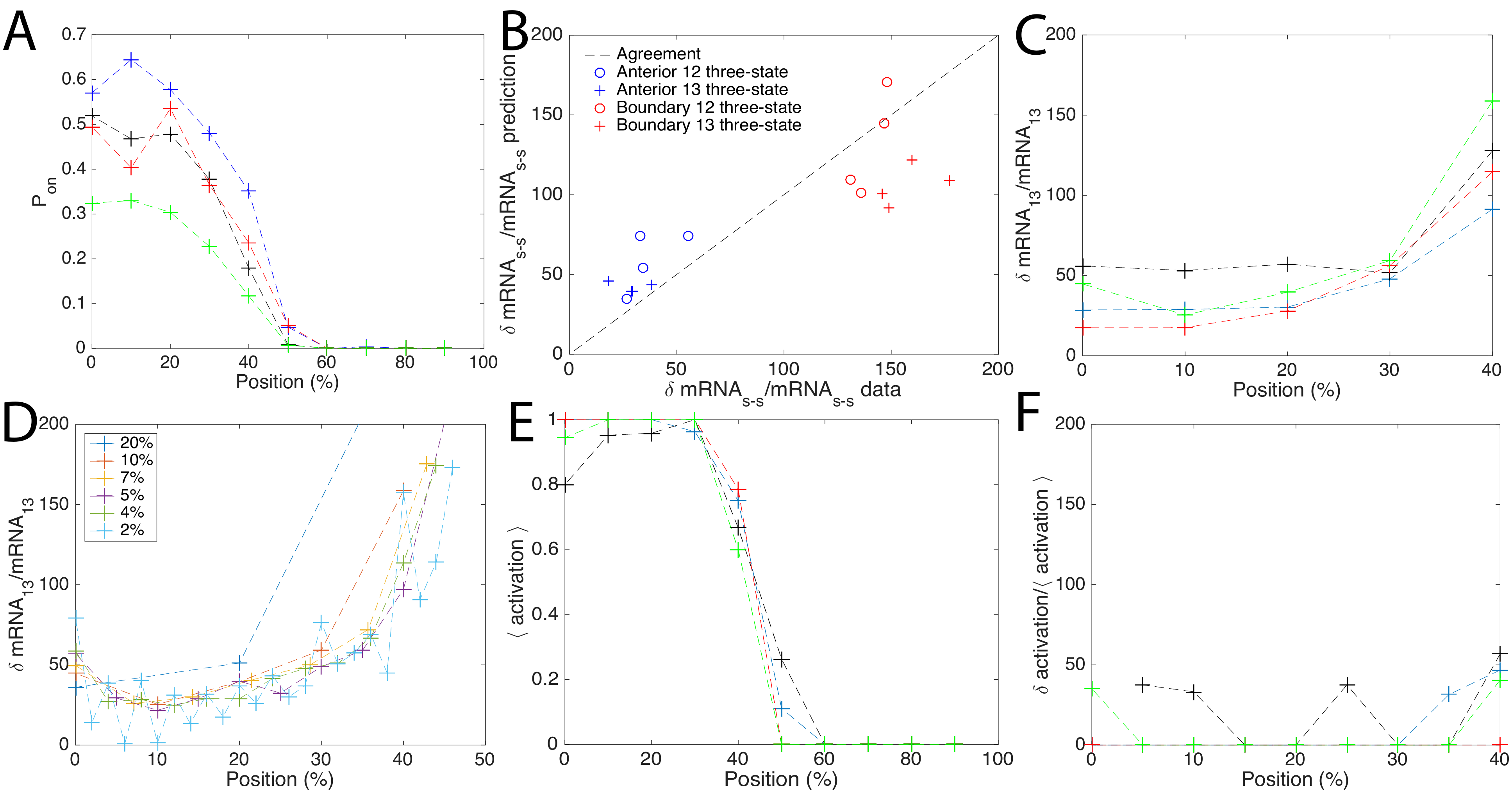

Supplement: S9 Fig — A. The mean probability of the gene to be ON at any time during the cell cycle as a function of the embryo length (binary approximation). B. Comparison of the relative error in the mRNA produced during the steady state of the interphase estimated empirically from data (abscissa) and from theoretical arguments in Eq. 62 using the inferred parameters from the autocorrelation function (ordinate), in the anterior (blue) and the boundary (red) regions, show very good agreement. C. The conclusions about precision do not depend on the embryo. The relative error of the total mRNA produced in cell cycle 13 as a function of position for windows equal to 10% of the embryo length. Each colored line represents one embryo. The same data plotted as an average over embryos with the variance as error bars is shown in Fig 7 of the main text. D. The conclusions about precision do not depend on the window size. The total mRNA produced in cell cycle 13 as a function of position for different window sizes. Except for very large scales (20%) and very small scales comparable to one nuclear width (2%, the relative error as a function of position is reproducible. E.The mean probability for the gene to be ON averaged over the cell cycle. F. The relative error of the discrete variable that describes the probability of the gene to be ON at any time during the cell cycle as function of position. The relative error is much lower in the anterior compared to the error in the total produced mRNA, but remains high at the boundary. In A, C, E and F each colored lines describe different embryos. (TIF) [file pcbi.1005256.s010.tif]

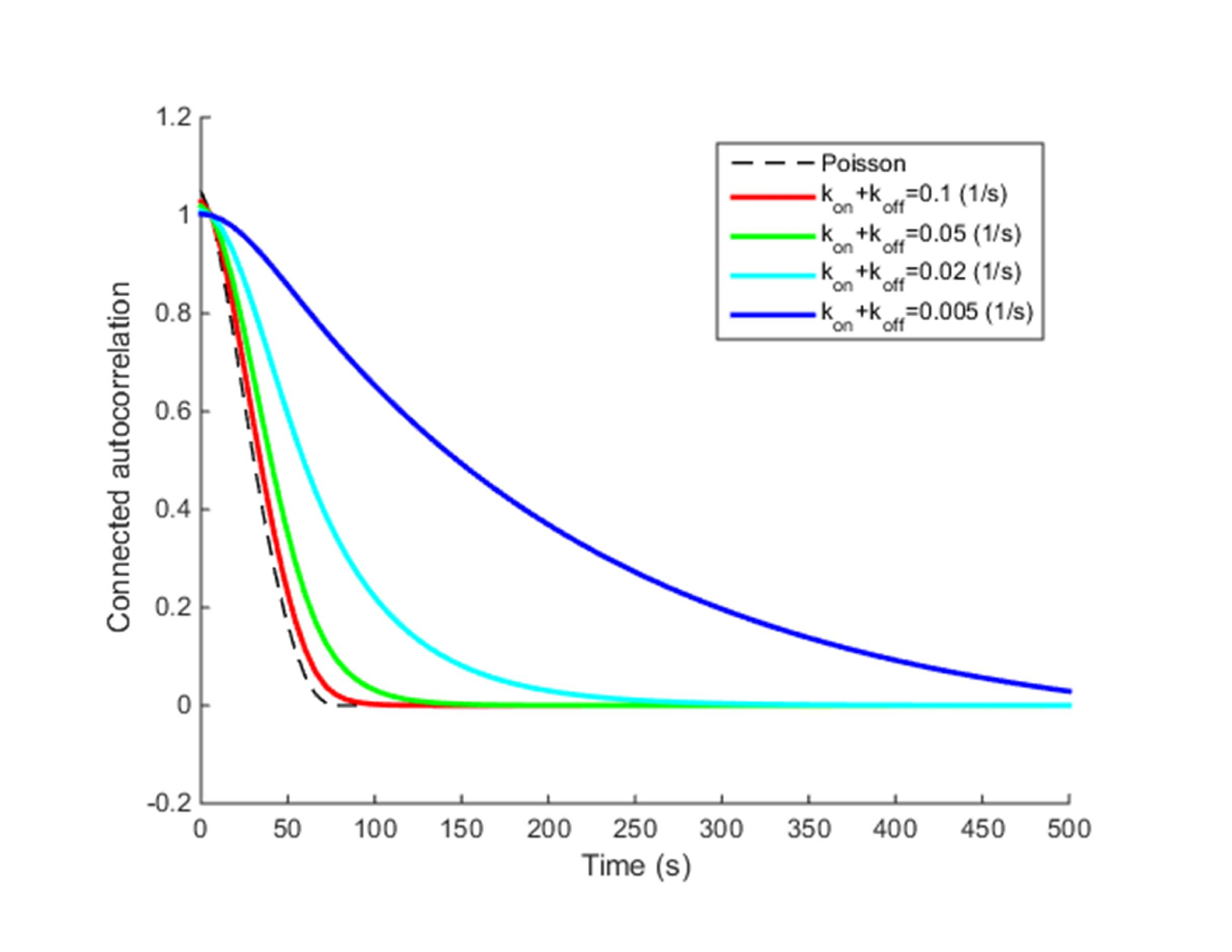

Supplement: S10 Fig — Autocorrelation functions of the Poisson-like model (dashed black) and the two-state models (solid) with Pon = 0.1 (similar to the inferred value in the boundary region) and varying kon + koff. In the inferred parameter regime (approximately green line), longer time traces do not help distinguish the two models based on the autocorrelation function. For large kon + koff values the shape of the autocorrelation function is dominated by the autocorrelation of the fluorescent probe and the Poisson-like and two state model autocorrelation functions look very similar, even for long traces. (TIF) [file pcbi.1005256.s011.tif]

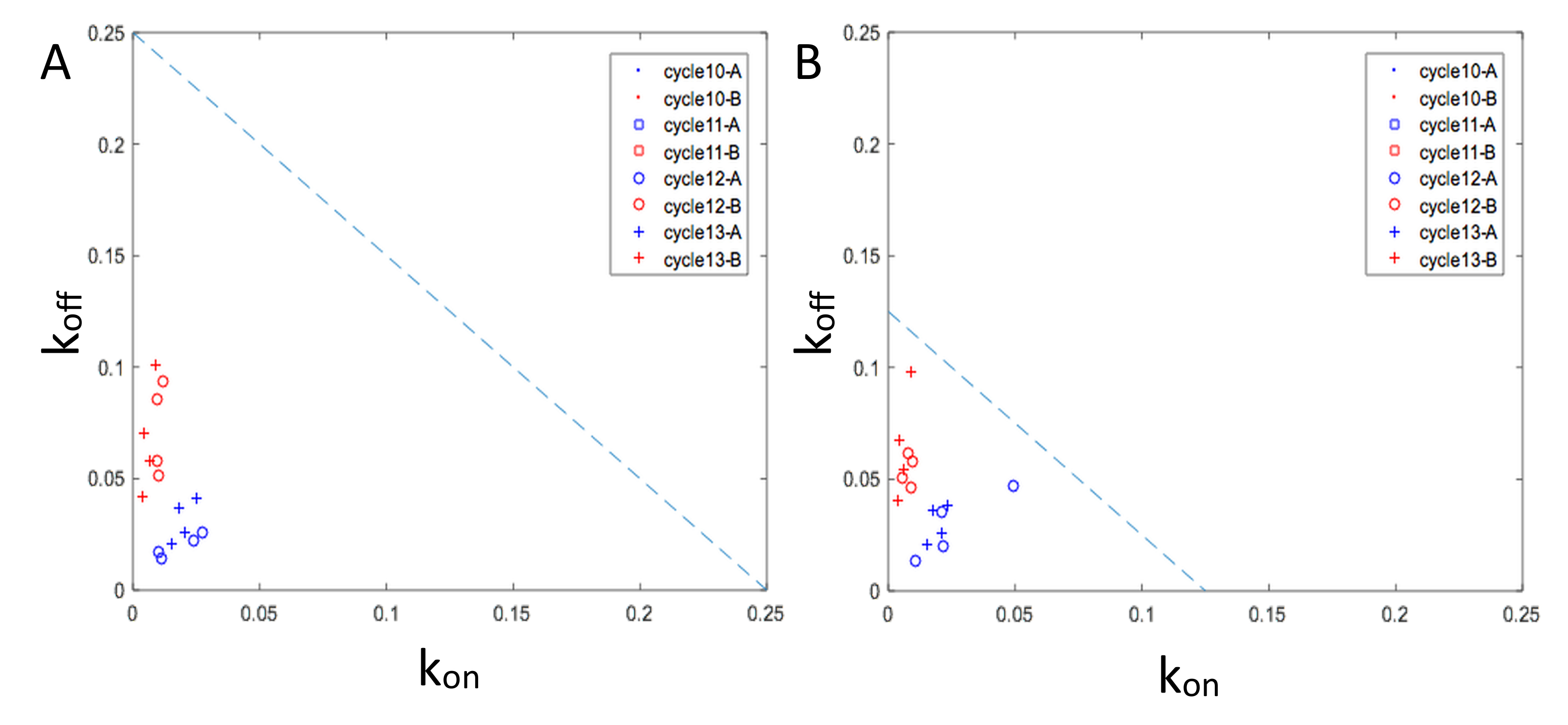

Supplement: S11 Fig — Assuming different buffering times for the polymerase does not strongly affect the fit of the switching rates: a fit with τblock = 4s (A) and τblock = 8s. τblock = 6s is used in the main text in Fig 5D. (TIF) [file pcbi.1005256.s012.tif]
